# Improved recovery of urinary small extracellular vesicles by differential ultracentrifugation

**DOI:** 10.1038/s41598-024-62783-9

**Published:** 2024-05-28

**Authors:** Ana Teixeira-Marques, Sara Monteiro-Reis, Diana Montezuma, Catarina Lourenço, Miguel Carlos Oliveira, Vera Constâncio, José Pedro Sequeira, Carina Carvalho-Maia, Rui Freitas, Elena S. Martens-Uzunova, M. Helena Vasconcelos, Rui Henrique, Carmen Jerónimo

**Affiliations:** 1 Cancer Biology and Epigenetics Group, Research Center of IPO Porto (CI-IPOP)/CI-IPOP@RISE (Health Research Network), Portuguese Oncology Institute of Porto (IPO Porto)/Porto Comprehensive Cancer Center Raquel Seruca (Porto.CCC), R. Dr. António Bernardino de Almeida, 4200-072 Porto, Portugal; 2 INEGI-LAETA, Faculty of Engineering, University of Porto, Campus FEUP, Rua Dr. Roberto Frias, 400, 4600-465 Porto, Portugal; 3 IMP Diagnostics, Praça do Bom Sucesso, 61, Sala 808, 4150-146 Porto, Portugal; 4 Doctoral Programme in Medical Sciences, ICBAS-School of Medicine and Biomedical Sciences-University of Porto, R. Jorge de Viterbo Ferreira 228, 4050-313 Porto, Portugal; 5 i3S-Instituto de Investigação e Inovação em Saúde, Universidade do Porto, R. Alfredo Allen 208, 4200-135 Porto, Portugal; 6 Doctoral Programme in Biomedical Sciences, ICBAS-School Medicine and Biomedical Sciences, University of Porto, R. Jorge de Viterbo Ferreira 228, 4050-313 Porto, Portugal; 7 Epigenomics Unit, Cancer Epigenomics, Translational Medical Oncology Group (ONCOMET), Health Research Institute of Santiago de Compostela (IDIS), University Clinical Hospital of Santiago (CHUS/SERGAS), Santiago de Compostela, Spain; 8 Department of Pathology, Portuguese Oncology Institute of Porto (IPO Porto), R. Dr. António Bernardino de Almeida, 4200-072 Porto, Portugal; 9 Department of Urology, Portuguese Oncology Institute of Porto (IPOPorto), R. Dr. António Bernardino de Almeida, 4200-072 Porto, Portugal; 10 Department of Urology, Erasmus MC Cancer Institute, University Medical Center Rotterdam, Be-331, PO Box 2040, 3000 CA Rotterdam, The Netherlands; 11 Cancer Drug Resistance Group, IPATIMUP-Institute of Molecular Pathology and Immunology, University of Porto, R. Alfredo Allen 208, 4200-135 Porto, Portugal; 12 Department of Biological Sciences, FFUP-Faculty of Pharmacy, University of Porto, R. Jorge de Viterbo Ferreira 228, 4050-313 Porto, Portugal; 13 Department of Pathology and Molecular Immunology, School of Medicine and Biomedical Sciences, University of Porto (ICBAS-UP), R. Jorge de Viterbo Ferreira 228, 4050-313 Porto, Portugal

**Keywords:** Tumour biomarkers, Tumour biomarkers, Isolation, separation and purification, Microbiology techniques

## Abstract

Extracellular vesicles (EVs) are lipid-membrane enclosed structures that are associated with several diseases, including those of genitourinary tract. Urine contains EVs derived from urinary tract cells. Owing to its non-invasive collection, urine represents a promising source of biomarkers for genitourinary disorders, including cancer. The most used method for urinary EVs separation is differential ultracentrifugation (UC), but current protocols lead to a significant loss of EVs hampering its efficiency. Moreover, UC protocols are labor-intensive, further limiting clinical application. Herein, we sought to optimize an UC protocol, reducing the time spent and improving small EVs (SEVs) yield. By testing different ultracentrifugation times at 200,000*g* to pellet SEVs, we found that 48 min and 60 min enabled increased SEVs recovery compared to 25 min. A step for pelleting large EVs (LEVs) was also evaluated and compared with filtering of the urine supernatant. We found that urine supernatant filtering resulted in a 1.7-fold increase on SEVs recovery, whereas washing steps resulted in a 0.5 fold-decrease on SEVs yield. Globally, the optimized UC protocol was shown to be more time efficient, recovering higher numbers of SEVs than Exoquick-TC (EXO). Furthermore, the optimized UC protocol preserved RNA quality and quantity, while reducing SEVs separation time.

## Introduction

Extracellular vesicles (EVs) are lipid-bound vesicles secreted by cells into the surrounding extracellular environment^1^. EVs may be classified into three main categories, based on their biogenesis: exosomes (40–200 nm diameter), microvesicles (100–1,000 nm diameter) and apoptotic bodies (100–5000 nm diameter)^2–4^. Exosomes are formed by the fusion of multivesicular bodies with the plasma membrane, whereas microvesicles are derived from budding directly from the plasma membrane^2^. In contrast, apoptotic bodies are shed from the plasma membrane of dying cells^2^. EVs incorporate various molecules from their cell of origin, including membrane receptors, proteins, nucleic acids, lipids and metabolites, which may be transported to local or distant cells through the circulation, affecting the activity of recipient cells^5^. Current separation techniques are unable to entirely separate each of these populations, due to size and density overlap. Therefore, we refer to them as large EVs (LEVs; > 200 nm diameter) or small EVs (SEVs; ≤ 200 nm diameter), as recommended by MISEV2018 guidelines^6^.

EVs may be separated from virtually all body fluids, such as blood, cerebrospinal fluid or urine^7^. Urine was shown to contain EVs derived from the different genitourinary organs, thus constituting a promising source of biomarkers for genitourinary tract diseases^8^. Urinary EVs may be separated using different methods. The most widely used method is differential ultracentrifugation (UC), followed by density ultracentrifugation (dUC) and precipitation, using commercially available kits such as Exoquick-TC® (EXO) (System Biosciences, CA, USA)^3^. The latest consists of a polymer that precipitates EVs by forming a network, which entraps all EVs between 30 and 200 nm in size^9^. UC and dUC protocols follow serial centrifugation steps to separate different EVs and other molecular components in the sample based on their differences in size and density. In addition to size and density, EV separation by ultracentrifugation also depends on the properties of the biofluid that contains the EVs, such as viscosity, density, and temperature^9,10^. Furthermore, dUC uses a density cushion or a gradient in addition to UC to obtain samples with increased purity^9,10^.

The UC procedure takes advantage of the aforementioned EV physical properties to segregate different EV populations from other sample components^11^. A typical UC protocol usually includes a low-speed centrifugation step (200–3500*g*) to remove cells and cell debris from the sample, followed by subsequent collection or removal of LEVs through an intermediate speed centrifugation (10,000–20,000*g*)^3,10^. To collect SEVs, a high-speed ultracentrifugation is then commonly performed (100,000–200,000*g*)^10,12^. To obtain cleaner samples, with less co-separated non-EV components, a washing step is generally carried out by resuspending the SEV pellet in PBS followed by another high-speed ultracentrifugation (100,000–200,000*g*)^10^.

**Figure 1:**
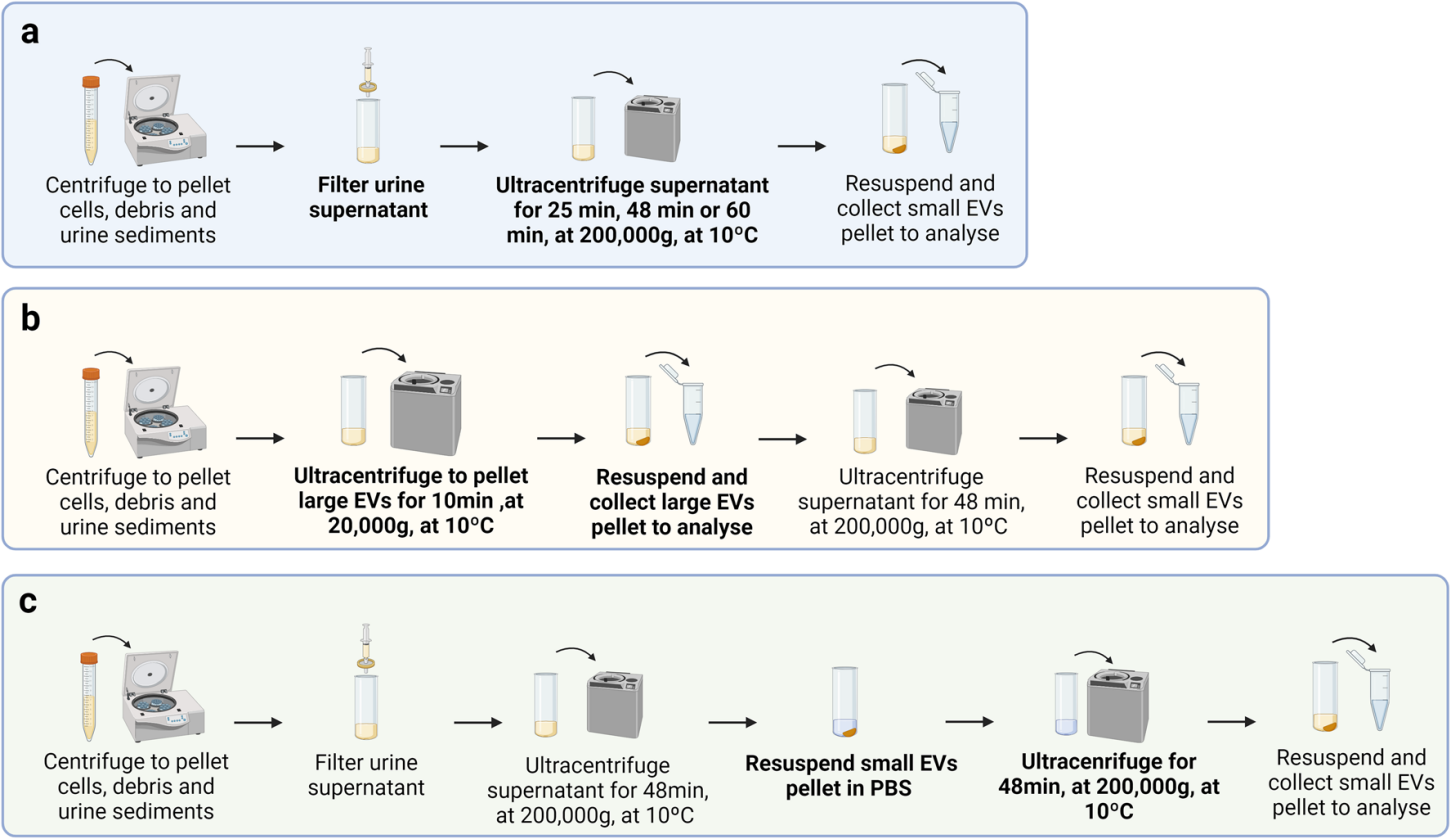
Workflow for each differential ultracentrifugation (UC) optimization protocol. (**a**) Protocols tested included 25 min (UC25min), 48 min (UC48min) and 60 min (UC60min) ultracentrifugation times, carried out to obtain small EVs (SEVs). (**b**) Protocol in which large EVs (UCLEVs) pelleting was performed. (**c**) Illustration from UC with washing step (UCwash). Figure was created with BioRender.com.

Although UC is an effective method for SEV separation, the technique is time-consuming and labor-intensive, limiting translational research^13^. Moreover, UC steps might result in limited recovery yield of SEVs^13^. Considering these shortcomings, we sought to optimize the standard UC protocol to facilitate its use in biomarker studies. For this, we varied different parameters of the standard UC protocol aiming to improve SEV recovery from urine (Fig. 1). Firstly, we compared different ultracentrifugation times, LEV pelleting versus filtering, and washing versus no wash step. Then, we compared SEV recovery and purity using the optimized UC protocol versus dUC and precipitation protocols. Finally, RNA amount and size distribution were assessed for the obtained SEVs using the optimized UC protocol and dUC.

## Results

### Different ultracentrifugation times affect SEVs’ recovery

The influence of different ultracentrifugation times on SEVs pelleting was assessed by performing ultracentrifugations with a duration of 25 min (UC25min), 48 min (UC48min) or 60 min (UC60min). Data that is represented was collected from three independent replicates. To establish the optimum ultracentrifugation time, we followed the protocol shown in Fig. 1a.

To establish ultracentrifugation times for testing, 2 different SEV densities were selected according to values reported in the literature. Based on maximum SEVs’ density of 1.18 g/cm^3^^14^, the time needed to theoretically pellet SEVs with at least 1.18 g/cm^3^ of a size equal or higher to 40 nm was calculated to be 19 min (Table 1). Presuming minimum EVs density of 1.08 g/cm^3^^15^ the calculated time was 42 min^16^. These times were extended by 6 min required for the ultracentrifuge to reach 200,000*g* (Table 1). A sedimentation cut-off size of 40 nm^2^ was assumed for both protocols since this is the reported minimum SEVs size. We presumed urine viscosity and density to be equal to water, i.e. viscosity of 1.3076 cP and density of 1.0 g/cm^3^ as assessed with the water density and viscosity calculators assuming a temperature of 10 °C (Table 1)^16–18^.

**Table 1:** Values used as input in the centrifugation parameters calculator to predict ultracentrifugation times for UC25min, UC48min and large EVs (LEVs) pelleting from (UCLEVs) protocol.

	Vesicles density [g/cm^3^]	Urine density [g/cm^3^]	Urine viscosity [cP]	Rotation speed [g]	Predicted centrifugation time [min]	Complete sedimentation cut-off size [nm]
UC25min	1.18	1.0	1.3076	200,000	19 + 6	40
UC48min	1.08	1.0	1.3076	200,000	42 + 6	40
UCLEVs (LEVs pelleting)	1.15	1.0	1.3076	20,000	8 + 2	210

Values for vesicles density^14,15,31^, urine density and viscosity^17,18^, predicted centrifugation time^16^ and complete sedimentation cut-off size^2^ are represented.

**Figure 2:**
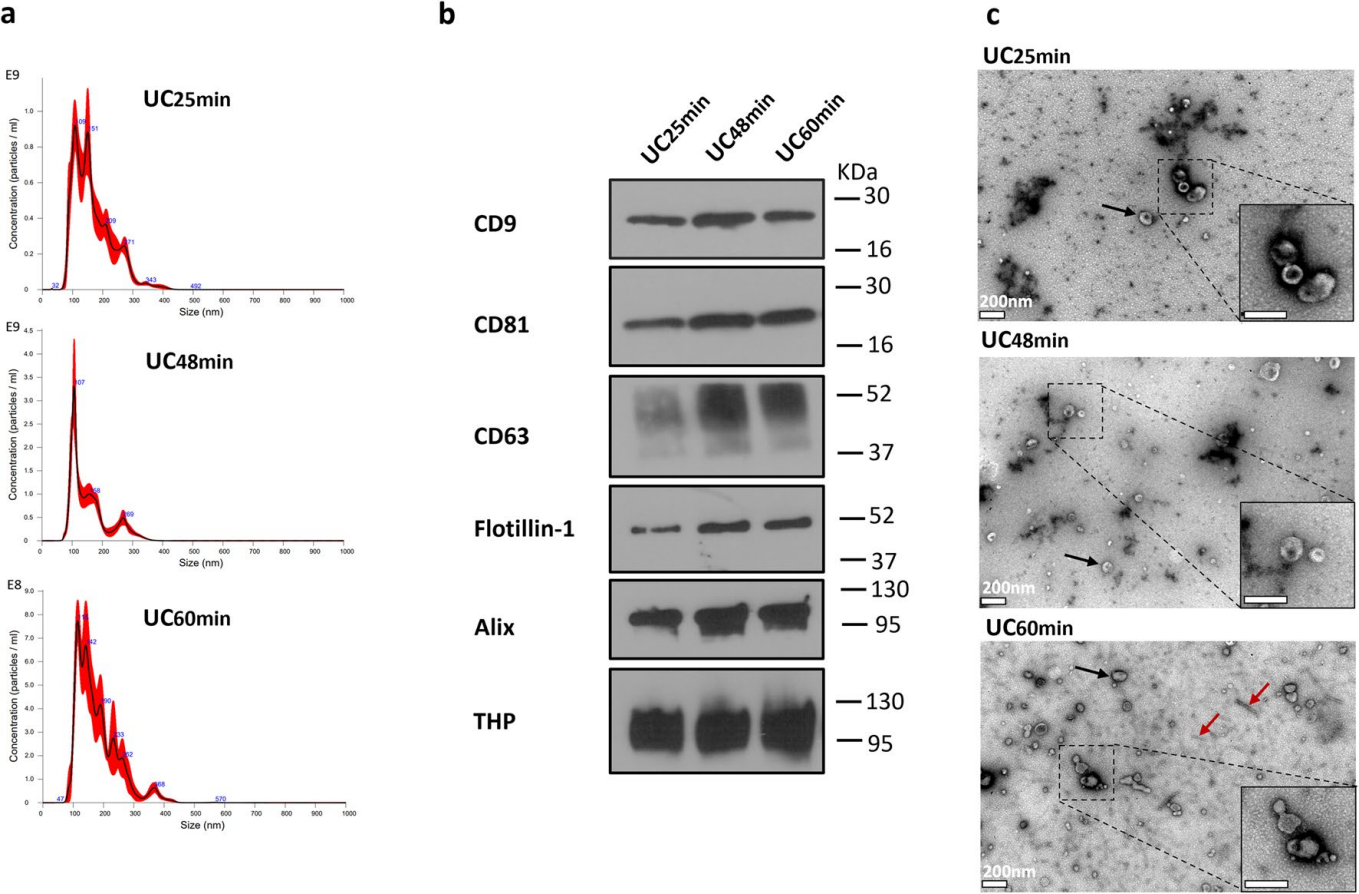
Characterization of small EVs (SEVs) isolated from 25 min (UC25min), 48 min (UC48min) and 60 min (UC60min) ultracentrifugation. (**a**) Nanoparticle tracking analysis (NTA) graphs display in y-axis: concentration (Particle/mL), and in x-axis: size (nm). (**b**) Western-blot (WB) for CD9, CD81, CD63, Flotillin-1, Alix and THP on SEVs samples. Western-blot images were cropped; the original blots are presented in Fig. S7 from Supplementary Material S2. (**c**) Transmission electron microscopy (TEM) analysis, in which black and red arrows correspond to SEVs and THP polymer (defined as the typical structure usually observed for THP protein), respectively. White bar represents a 200 nm scale. NTA and WB techniques were performed in 3 urine samples (only one sample is represented), and TEM in one sample.

Subsequent Nanoparticle Tracking Analysis (NTA) revealed that compared to UC25min, the UC48min and UC60min protocols achieved a 1.6 and 1.2 fold-increase in particle concentration, respectively (Fig. 2a; Table S2 and Fig. S1a—Supplementary Material S1). CD9, CD81, and Tamm–Horsfall protein (THP) were detected by Western-blot independently of the ultracentrifugation time, whereas CD63, Flotillin-1, and Alix were mostly present in UC48min and UC60min (Fig. 2b; Fig. S1—Supplementary Material S2). Furthermore, the highest protein concentration was obtained with UC60min, followed by UC48min and UC25min (Table S2 and Fig. S1—Supplementary Material S1). Transmission electron microscopy (TEM) analysis showed SEV-like structures in all three conditions UC25min, UC48min, and UC60min (Fig. 2c), but UC60min disclosed more THP polymers (defined as THP protein structures typical observed by electron microscopy), which is in line with the increase of protein concentration detected by microBCA (Fig. 2c; Table S2—Supplementary Material S1).

### Urine supernatant filtering and pelleting of LEVs influence SEVs’ concentration and size

LEVs pelleting is a common practice in UC protocols, although this step was shown to decrease SEVs recovery^16^. Thus, we compared the effect of LEVs pelleting by ultracentrifugation (UCLEVs) on SEVs recovery, with the use of urine supernatant filtering as a replacement of LEVs pelleting by ultracentrifugation (UC48min) (Fig. 1a,b).

**Figure 3:**
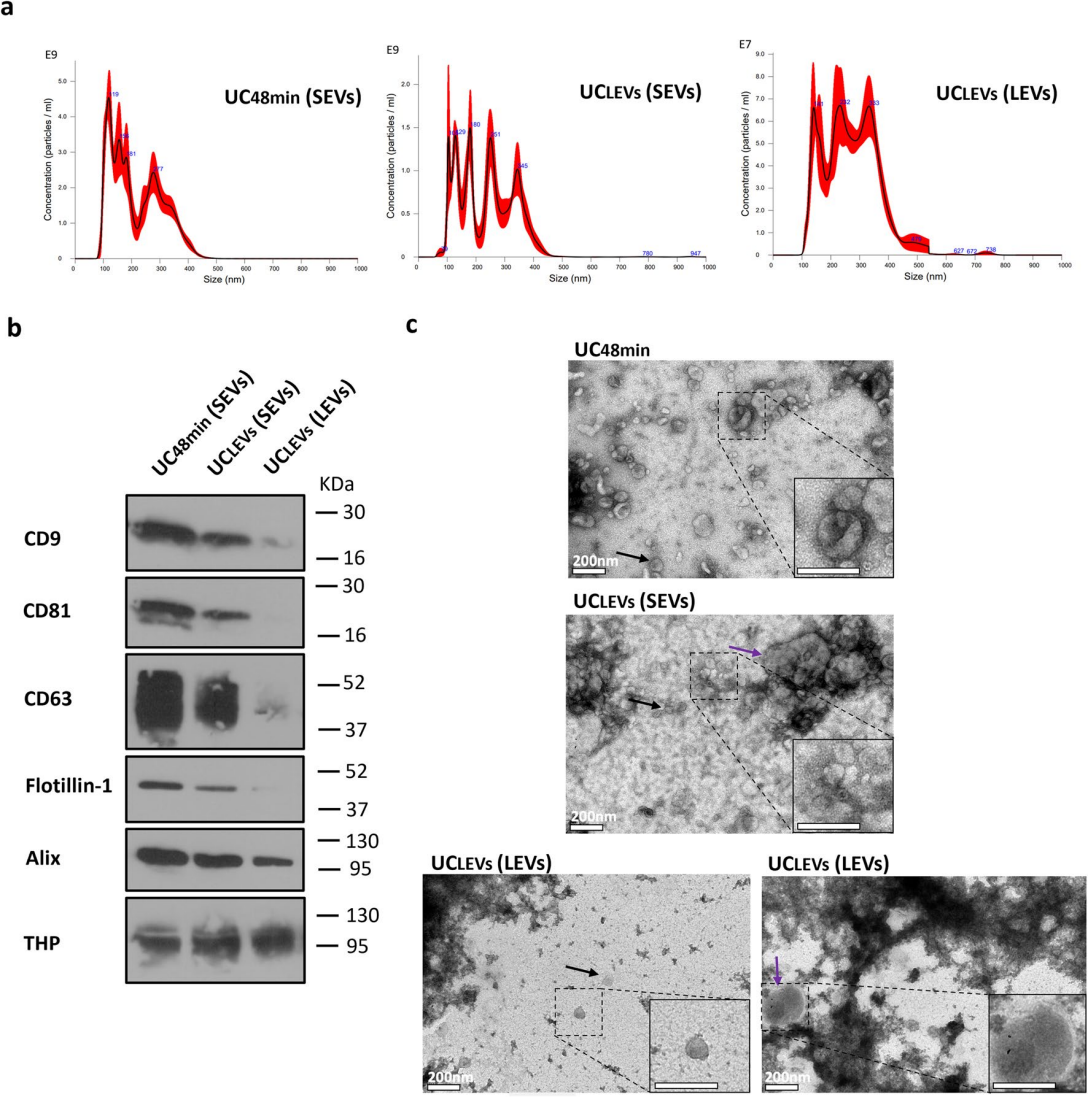
Characterization from small (SEVs) and large EVs (LEVs) pellet comparing LEVs (LEVs) pelleting (UCLEVs) with urine supernatant filtering (UC48min). (**a**) Nanoparticle tracking analysis (NTA) graphics, in which y axis represents particle concentration (Particle/mL) and x-axis the size (nm). (**b**) Western-blot (WB) with antibodies against CD9, CD81, CD63, Flotillin-1, Alix and THP. Western-blot images were cropped; the original blots are presented in Fig. S8 from Supplementary Material S2. (**c**) Transmission electron microscopy (TEM) images from EVs samples, in which black and purple arrows indicates SEVs and LEVs respectively. White bar corresponds to a 200 nm scale. NTA and WB techniques were performed in 3 urine samples (only one sample is represented), and TEM in one sample.

When using the UCLEVs protocol, the SEVs pellet contained 2.7 times more particles with a lower mean size compared to the LEVs pellet, suggesting that SEVs pellet is enriched in small particles (Fig. 3a; Table S3 and Fig. S1—Supplementary Material S1). Nevertheless, TEM analysis revealed that SEVs were present in the LEVs pellet and that LEVs were observed in the SEVs pellet (Fig. 3c).

Comparison of the SEVs pellets obtained by UCLEVs or UC48min demonstrated that UC48min recovered SEV pellets with 1.7-fold higher particle concentration (Fig. 3a; Table S3 and Fig. S1—Supplementary Material S1). Furthermore, EV-specific markers were consistently detected only in SEVs pellets separated with UC48min (Fig. 3b; Fig. S2—Supplementary Material S1). TEM analysis demonstrated that characteristic SEVs were mostly observed in UC48min, whereas a mixture of SEVs and LEVs were seen in UCLEVs (Fig. 3c).

### Washing step strongly impacts SEVs’ recovery

**Figure 4:**
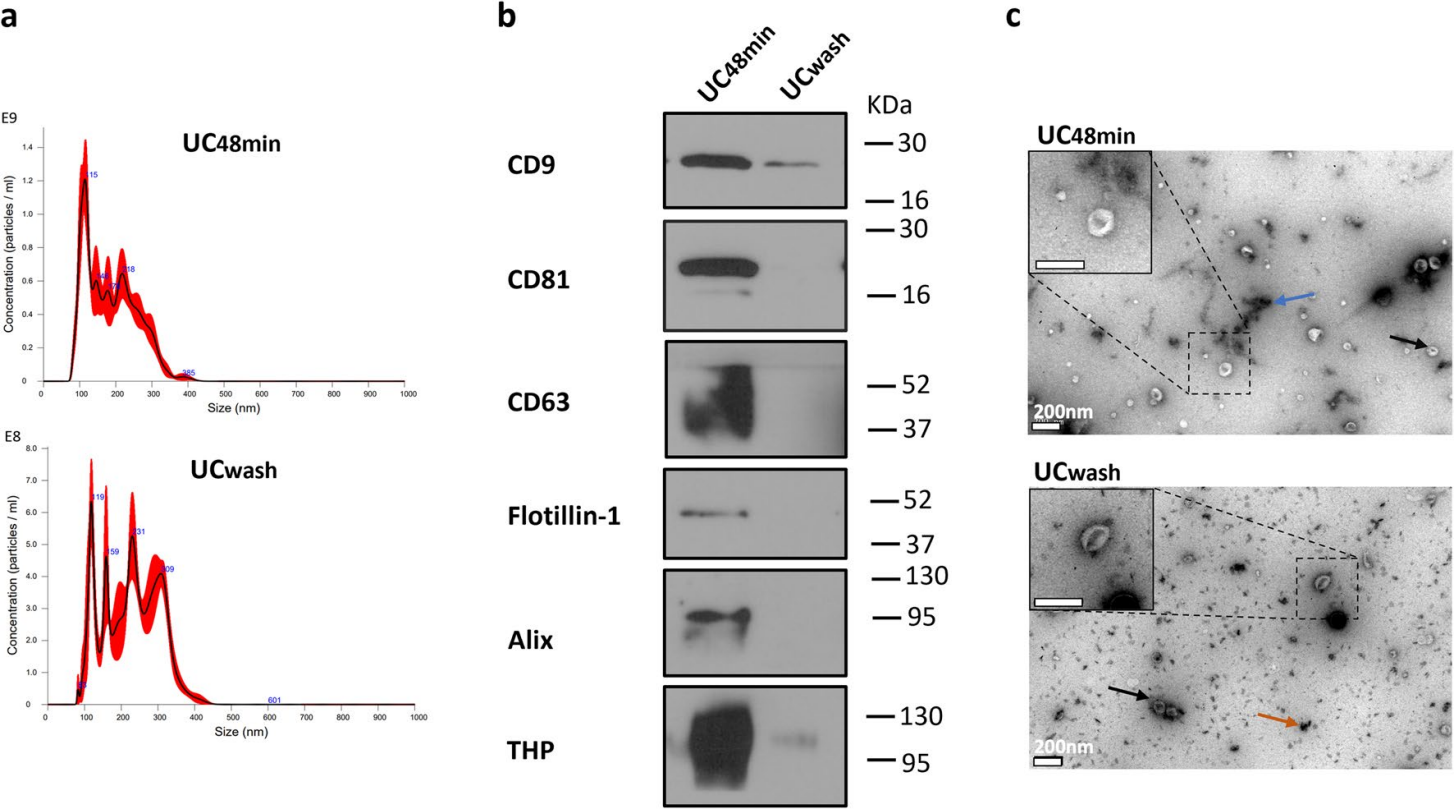
Characterization of small EVs (SEVs) from protocols without (UC48min) and with washing step (UCwash). (**a**) Nanoparticle tracking analysis (NTA) graphic where x-axis represents particle size distribution (nm) and y-axis concentration (Particle/mL). (**b**) Western-blot (WB) for common EV markers including CD9, CD81, CD63, Flotillin-1 and Alix and for THP contaminant. Western-blot images were cropped; the original blots are presented in Fig. S7 from Supplementary Material S2. (**c**) Transmission electron microscopy (TEM) images from SEVs, in which black, blue, and orange arrows point out SEVs, THP-like polymer and protein precipitate, respectively. White bar indicates a 200 nm scale. NTA and WB techniques were performed in 3 urine samples (only one sample is represented), and TEM in one sample.

To investigate to what extent a SEV washing step reduces SEVs recovery, we compared SEVs recovery obtained with and without washing (Fig. 1c). Washing with PBS led to 2.1 decrease in particle concentration and higher particle mean size demonstrating that SEV washing reduces small particle recovery (Fig. 4a; Table S4 and Fig. S1—Supplementary Material S1). Consistently, the protein concentration of the UCwash pellet was lower than that of UC48min, although with a higher purity ratio (Table S4 and Fig. S1—Supplementary Material S1). Furthermore, detection of EV-classical proteins was hampered in the UCwash pellet (Fig. 4b; Fig. S1—Supplementary Material S1). In TEM analysis UCwash presented fewer SEVs and more debris than UC48min protocol (Fig. 4c).

### The choice of separation method affects SEVs’ yield

**Figure 5:**
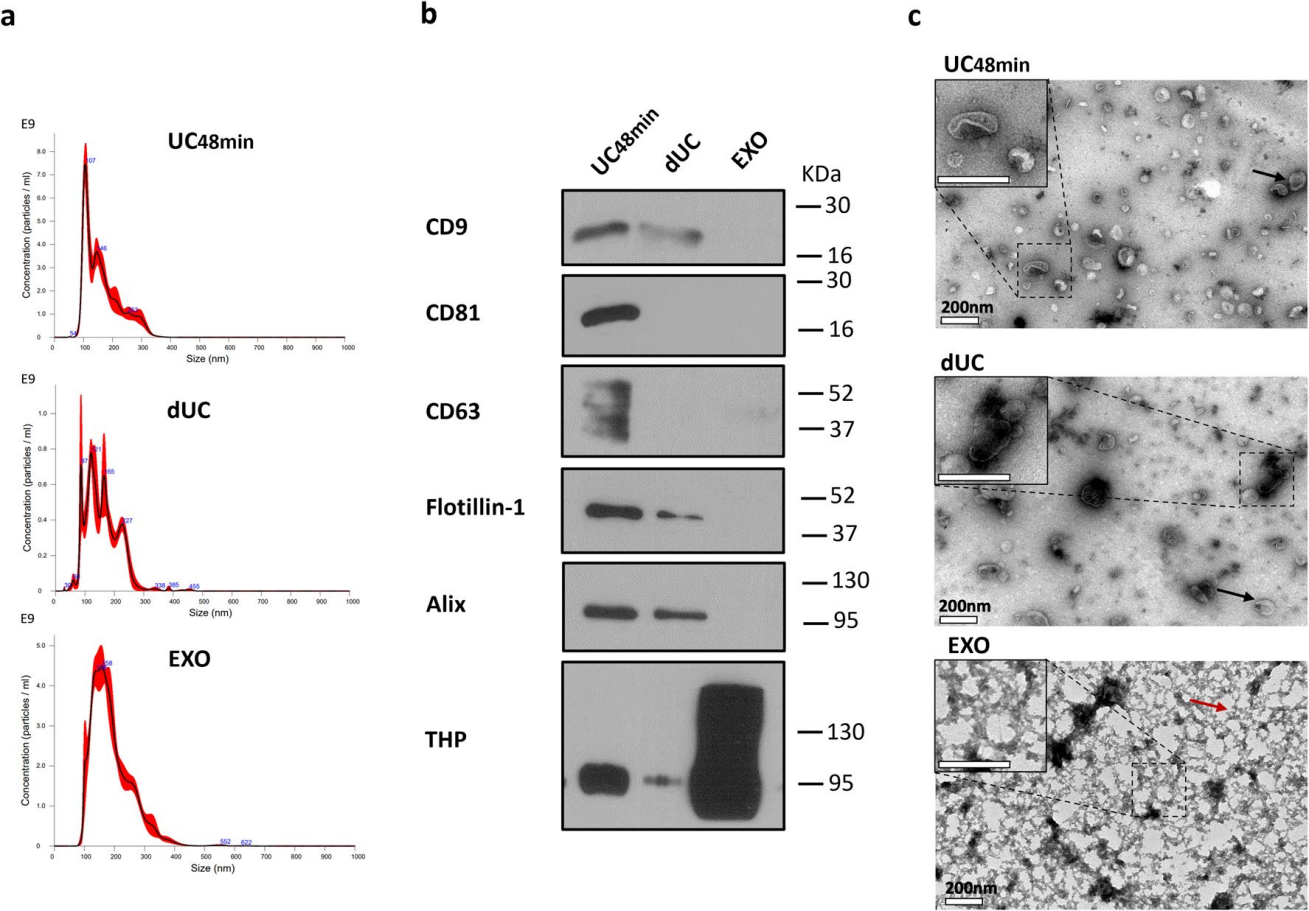
Comparison between optimized differential ultracentrifugation (UC48min), density ultracentrifugation (dUC) and Exoquick (EXO) separation methods. (**a**) Nanoparticle tracking analysis (NTA) graphic represents particle size distribution in x-axis (nm) and concentration in y-axis (particle/mL). (**b**) Western-blot (WB) signals obtained from CD9, CD81, CD63, Flotillin-1, Alix and THP markers. Western-blot images were cropped; the original blots are presented in Fig. S9 from Supplementary Material S2. (**c**) Transmission electron microscopy (TEM) micrographs, in which the scale bar corresponds to 200 nm. Black and red arrows indicate small EVs (SEVs) and EXO polymer, respectively. NTA and WB techniques were performed in 3 urine samples (only one sample is represented), and TEM in one sample.

Next, we compared the performance of a precipitation technique with a density UC protocol (dUC) and the UC48min protocol. SEV separation using the EXO kit yielded higher particle concentration than dUC (Fig. 5a; Table S5 and Fig. S1—Supplementary Material S1). However, following the EXO protocol no EV markers were detected except Alix (detected in 1 sample), contrary to dUC, which obtained SEVs that were positive for CD9, Flotillin-1, and Alix (Fig. 5b; Table S5—Supplementary Material S1). Particle concentration of SEVs separated with the EXO and UC48min protocols was comparable (1.03 fold-difference) and 1.9-fold higher than the one measured after dUC (Fig. 5a; Table S5 and Fig. S1—Supplementary Material S1). Furthermore, UC48min SEVs were enriched in EV markers, testing positive for CD81 and CD63 in addition to CD9, Alix and Flotillin-1 (Fig. 5b). THP was strikingly abundant in SEVs isolated with the EXO protocol, and almost absent in dUC, which is in line with protein concentration results (Fig. 5b; Table S5 and Fig. S1—Supplementary Material S1) and purity ratios, suggesting that dUC is the method that provides highest purity (Table S5 and Fig. S1—Supplementary Material S1). Importantly, TEM analysis revealed EV-typical structures in UC48min and dUC protocols, whereas it was not possible to detect SEVs in the EXO pellet, possibly due to the presence of Exoquick polymer (Fig. 5c). Since UC48min and dUC were demonstrated to be the methods with increased SEV recovery and purity, respectively, SEVs from 3 more patients were separated using those methods. Indeed, UC48min resulted in increased particle recovery as well as the expression of EV-classical markers (Fig. S3—Supplementary Material S1). Moreover, Cytochrome C was absent in both methods, while THP was mostly present in UC48min (Fig. S3—Supplementary Material S1). As expected, the purity ratio was increased in dUC compared with UC48min (Table S6 and Fig. S3—Supplementary Material S1).

Due to THP contamination of UC48min compared with dUC, a preliminary test with NaCl and 2-mercaptoethanol treatment was performed to evaluate the potential influence of THP in SEVs yield. NTA analysis revealed that particle recovery was not improved by both treatments (Fig. S4, Methods S1 and S2—Supplementary Material S1), which might be explained by the fact that THP is not affecting SEVs recovery.

### UC48min and dUC protocol isolates SEVs’ enriched in small non-coding RNAs

**Figure 6:**
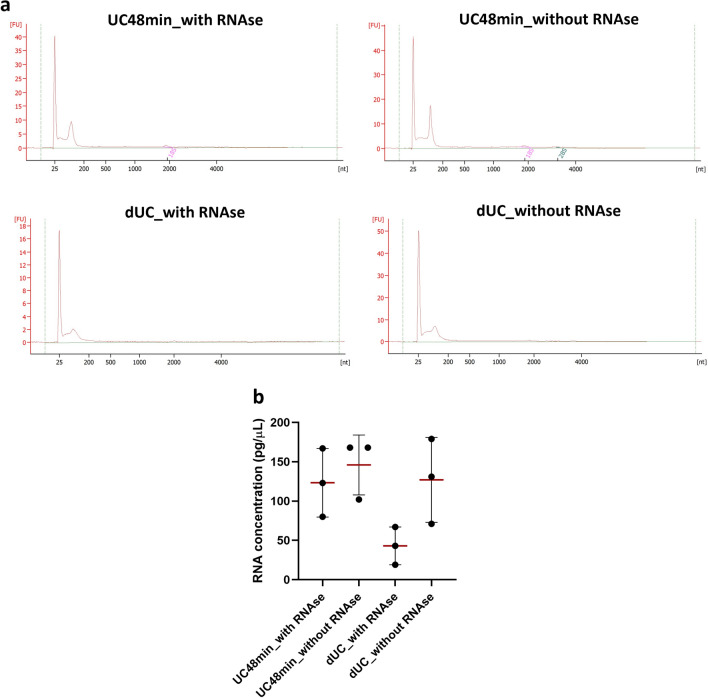
Bioanalyzer graphics representing RNA size distribution of UC48min and dUC in RNAse treated and non-treated samples and dot plots with standard deviation from RNA concentration. (**a**) Bioanalyzer graphs: y-axis corresponds to fluorescence units (FU) and x-axis to the number of nucleotides (nt). (**b**) Horizontal red lines correspond to the means of RNA concentration of tested samples. Comparisons were made in 3 independent urine samples.

Bioanalyzer electropherograms demonstrated the presence of RNAs up to ~ 200nt in UC48min and dUC-separated SEVs (Fig. 6a; Fig. S5—Supplementary Material S1). To evaluate the RNA purity of UC48min and dUC, conditions with and without RNAse were tested. Indeed, there was a 1.18-fold increase in UC48min without RNAse compared with RNAse, although the bioanalyzer shows a similar profile in both conditions (Fig. 6b; Fig. S5—Supplementary Material S1). Additionally, a 2.95-fold increase was achieved for dUC samples treated with RNAse compared without RNAse condition (Fig. 6b; Table S7 and Fig. S5—Supplementary Material S1). UC48min with RNAse achieved a 2.87-fold increase in RNA concentration compared with dUC (Fig. 6b; Table S7 and Fig. S5—Supplementary Material S1). Additionally, RNAse treatment in dUC resulted in a striking decrease of ~ 25nt peak compared with the condition without treatment (Fig. 6a; Fig. S5—Supplementary Material S1).

### UC48min field performance

**Figure 7:**
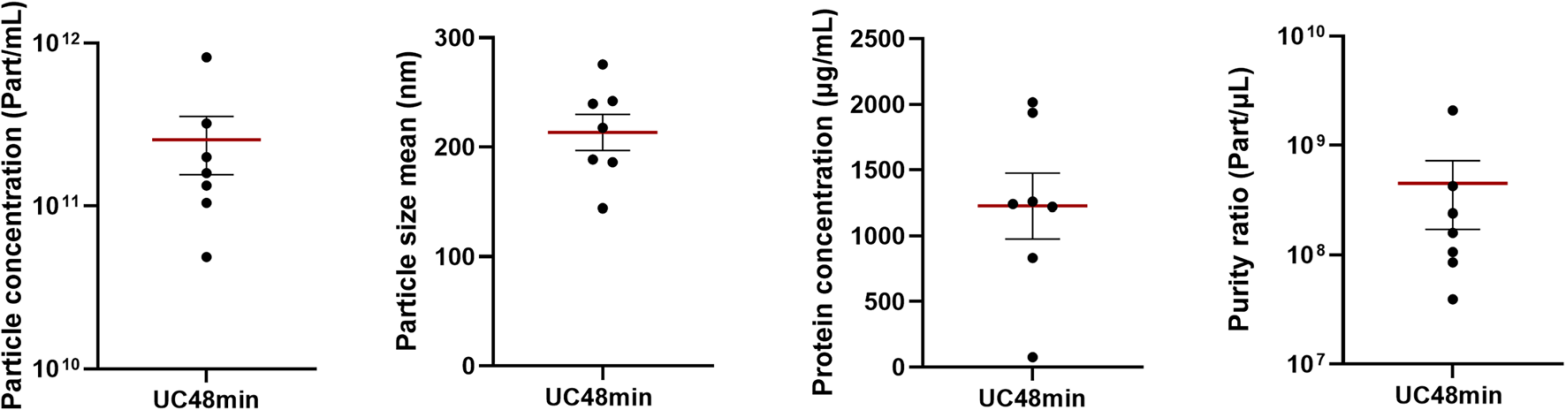
Dot plots with standard deviation from particle concentration, particle mean size, protein concentration and purity ratio among the tested samples using UC48min protocol. Horizontal red lines represent the means of tested samples. All parameters were measured in seven samples.

To test the reproducibility of UC48min protocol, seven samples (3 healthy donors and 4 cancer patients) were isolated and characterized by NTA, microBCA and WB. Mean particle and protein concentration were 2.55E+11 part/mL and 1226.0 µg/mL, respectively (Fig. 7; Table S8 and Fig. S6—Supplementary Material S1). At least one transmembrane (CD9, CD81 or CD63) and one cytosolic marker (Alix or Flotillin-1) were detected in all tested samples (Fig. S6—Supplementary Material S1). Moreover, THP was present in six samples, whereas Cytochrome C and Lamin A/C, which are associated with intracellular compartments, were not detected (Fig. S6—Supplementary Material S1). Additionally, to test the reproducibility of UC48min method regarding RNA yield performance, SEV-RNA was extracted from 16 samples (3 healthy donors and 13 cancer patients). A mean RNA concentration of 316 pg/µL was obtained (from 130 to 1571 pg/µL) and small RNA between ~ 25 nt and 200 nt were present in all samples (Fig. S7—Supplementary Material S1).

## Discussion

Urinary EVs provide a source of different molecules that may be used as biomarkers for genitourinary diseases, including cancer. UC is the most commonly used protocol for urinary SEV separation but is time-consuming and frequently results in significant SEV loss, which hampers the usage of this method for translational research. To overcome the limitations, here, we tested different settings for the two main steps of the UC protocol for SEV separation.

First, we compared the effect of different ultracentrifugation times for the 200,000*g* ultracentrifugation step, which pellets SEVs. Ultracentrifugation times of 25 min (UC25min) and 48 min (UC48min) were calculated using the publicly available Centrifugation Parameters Calculator, and 60 min (UC60min), was based on available literature^16,19–24^. The UC48min and UC60min protocols provided higher particle recovery compared to UC25min. This suggests that UC25min results in incomplete SEVs sedimentation, namely of lower density SEVs, since this time was calculated to pellet SEVs equal to or higher than 1.18 g/cm^3^. Indeed, these results demonstrate a consistency of the theoretical analysis performed by the Centrifugation Parameters Calculator. Although UC48min recovered higher particle concentration than UC60min, WB for EV-markers did not show strong differences between both protocols and appeared reduced when compared to UC48min. Additionally, increased protein concentration but not particle concentration in UC60min might be consistent with increased THP contamination compared with the other UC times. Importantly, longer ultracentrifugation times led to higher protein concentration, although including more contaminating proteins that precipitate along with longer ultracentrifugation times, as demonstrated by other research teams^25,26^. Thereby, UC48min was found sufficient and preferred to recover at least as many SEVs as UC60min.

Next, we tested the impact of the intermediate step, which includes either pelleting by UC (UCLEVs) or filtering of the urine supernatant (UC48min) to remove LEVs. It is noteworthy that, even when considering the complete sedimentation cut-off size of 210 nm to pellet LEVs, the Centrifugation Parameters Calculator predicted that 7–61% of SEVs between 50 and 150 nm would still be kept in the respective pellet. In fact, LEVs pelleting step decreased overall SEVs recovery, probably because SEVs were pelleted with LEVs, as observed in TEM images. Similar results have been also demonstrated by Bobrie et al.^27^. During short, low velocity ultracentrifugation (i.e. 10 min at 20,000*g*), SEVs that started their migration close to the pellet, may sediment simultaneously with LEVs that travel longer distances^16^. In contrast, LEVs placed close to fluid meniscus may experience insufficient time to sediment, and remain in the supernatant, being pelleted together with SEVs in subsequent ultracentrifugation steps at higher speed^16^. As seen in TEM analysis, LEVs were found in the SEVs pellet. Moreover, salts were macroscopically observed in the SEVs pellet (from UCLEVs protocol), which we hypothesize to precipitate at higher ultracentrifugation speeds and influence SEVs pelleting due to its reduced affinity with ultracentrifuge tube plastics, and because salts are denser than SEVs^28,29^. Consequently, salts may pellet before SEVs during ultracentrifugation, thus reducing SEVs adherence to the tubes, further decreasing SEVs recovery. Indeed, our TEM analysis of UCLEVs samples revealed images with overall poor quality due to high background noise, which has been also reported by Rikkert et al*.,* in samples with salts^30^. Additionally, EV-markers were consistently present in all tested samples of UC48min, while the expression was not detected in UCLEVs’ SEVs pellet in 2 of the 3 tested samples (Fig. S2—Supplementary Material S1). This might be because the number of SEVs obtained in UCLEVs was limited to be detected in WB, since TEM analysis showed typical EV structures. Hence, filtering urine supernatant instead of performing LEVs pelleting is the best strategy to obtain improved SEVs’ recovery as well as purity.

Additionally, we evaluated the effect of washing after a high-speed ultracentrifugation to obtain SEVs. Although the washing step resulted in higher purity ratio and lower THP contamination, a considerable reduction in SEVs recovery yield was attained. Whereas THP polymers were present in the protocol lacking the washing step, a considerable amount of protein precipitates was formed, as observed by TEM. This indicates that repeating high velocity ultracentrifugation steps, required for washing, precipitates proteins. Moreover, the washing step increased particle size, while reducing EVs recovery, indicating a loss of the smaller EV fraction. Thus, washing resulted in higher SEVs purity, but reduced SEVs recovery. Indeed, most authors usually perform this step to reduce possible non-EV protein content contamination. Hence, one should consider the final use of the separated SEVs. Regarding biomarker research, a higher recovery yield is preferable to avoid information loss, particularly when biofluid volume is limited.

The performance of the optimized UC48min protocol was compared with dUC and EXO in terms of recovery and purity. The UC48min protocol recovered all tested EV markers, namely CD9, CD81, CD63, Flotillin-1 and Alix, whereas EXO SEVs did not disclose any of these markers except Alix, which was present in only 1 sample. Importantly, and contrarily to the other methods, no SEVs were observed in EXO samples using TEM, whereas the polymer network was clearly visible in the acquired images. Intriguingly, particle concentration of EXO protocol was very similar to UC48min protocol, meaning that NTA was probably counting Exoquick polymer particles (observed in TEM images). Moreover, EXO SEVs displayed the highest THP and protein content with a very poor purity ratio, in line with those reported by Deun et al.^26^. Although dUC provided SEVs with the highest purity ratio regarding protein contamination, the yield was strikingly lower than the UC48min method and depicted reduced expression of EV-protein markers. Indeed, dUC is reported to lead to an increased purity ratio, which is of utmost importance when the downstream application concerns functional studies^6^.

Since UC48min and dUC enabled improved SEVs’ recovery and purity ratio, respectively, RNA was extracted from SEVs separated with these methods. RNA analysis by Bioanalyzer showed that UC48min and dUC are suitable for small non-coding RNA isolation since RNA up to ~ 200 nt was found to be enriched in SEV samples separated with those methods. However, RNA concentration was higher in the UC48min method. Although dUC resulted in samples with decreased THP protein, increased external RNA contamination, mainly of small RNAs of ~ 25 nt size, was attained compared with UC48min, which was proved by RNAse treated and non-treated conditions. This might be explained by the fact that the dUC protocol uses a long ultracentrifugation time (16h), which might lead to increased deposition of smaller molecules. Thus, to separate SEVs for biomarker research, UC48min seems to be a suitable method, since enables high SEV and RNA recovery in a shorter time.

Finally, the field performance of the UC48min protocol was evaluated using different patients. Among the tested samples (n = 7), at least one transmembrane and one cytosolic marker were detected, demonstrating that the UC48min protocol isolated SEVs from all tested samples. Although THP was detected in 6 out of the 7 tested samples, intracellular compartment components (Cytochrome C and Lamin A/C) were not detected demonstrating no contamination with cell debris components. Additionally, RNA was successfully extracted from all patients (n = 16), with small RNAs between ~ 25 nt and 200 nt being detected in all analyzed samples. Overall, the UC48min protocol demonstrated to result in consistent and reproducible protein and RNA analyses.

## Conclusion

Overall, we were able to design a 2h 15 min protocol that produces a urinary SEV isolate of adequate yield for translational research using 10 mL of − 80 °C frozen-stored supernatants. The presence of RNA components in the separated SEVs shows that this optimized UC protocol can be used for small non-coding RNA biomarker research. Validation in biomarker studies using urine samples of larger patient cohorts is envisaged for the near future.

## Materials and methods

### Urine collection

Samples from 9 healthy donors and 29 cancer patients with an age range of 18–87 years old, were collected from all patients and healthy donors after informed consent. Clinicopathologic data is presented in Table S1 from Supplementary Material S1. This study was approved by the institutional review board (Comissão de Ética para a Saúde) of the Portuguese Oncology Institute of Porto (CES_221/020). All procedures involving human participants were under the ethical standards of the institutional and/or national research committee and with the 1964 Helsinki declaration.

### EVs’ separation

Voided urine was collected and centrifuged (up to 50 mL of urine) at 3700*g* (in a Gyrozen 1580R centrifuge; Gyrozen, St Petersburg, Russia) during 20 min, at 4 °C to pellet cells and debris. The resultant supernatant was decanted and stored in 50 mL polypropylene tubes (Nerbe plus; Winsen, Germany) at − 80 °C until further use.

For the different separation procedures, the same samples were used for the compared conditions, e.g. for EV separation of UC time testing, aliquots of the same samples (3 samples per each tested condition) were used for the three ultracentrifugation times tested. This allowed us to overcome the bias associated with heterogeneity that might be presented among biological samples. All EV separation procedures were tested for the same volume of urine, of 10 mL.

### EVs' separation by ultracentrifugation (UC)

After thawing, 10 mL urine supernatant from each patient were vortexed to disperse EVs and protein aggregates. Then, centrifugation at 3000*g* during 15 min at 4 °C in a Sigma 3-16PK centrifuge (Sigma, Missouri, USA) was performed to pellet and discard urine sediment remains.

Ultracentrifugation steps described in this manuscript were performed in an Optima XE-100 Ultracentrifuge (Beckman Coulter, California, EUA) using the 70Ti rotor (Beckman Coulter, California, EUA) at 20,000*g*, 100,000*g* and 200,000*g* and thick wall polycarbonate tubes (Ref: 355631, Beckman Coulter, California, EUA.) with diameter: 25 mm, length: 89 mm and volume: 32 mL.

#### UC time selection: UC25 min, UC48 min, and UC60 min protocols

Ultracentrifugation times to pellet SEVs were calculated by the Centrifugation Parameters Calculator created by Livshits et al., using previously reported SEVs density and size as input values (described in Table 1)^14,15^. Urine viscosity and density were required parameters for the calculation of ultracentrifugation times. However, urine samples can vary a lot in composition and concentration adding the fact that currently there are no reported values of urine density and viscosity at 10°C. Thus, since urine is composed by 91–96% of water, values of water density and viscosity were used as an input in the Centrifugation Parameters calculator (Values described in Table 1)^17,18,28^. For comparison a UC protocol based on the literature, with a 60 min 200,000*g* ultracentrifugation was performed, to pellet SEVs from urine^19–24^.

#### Sample preparation and UC time testing

Urine supernatant was filtered through a PVDF filter (pore size 0.22 µm, Millipore, MA, USA). Supernatant aliquots were ultracentrifuged using 70Ti rotor at 200,000*g* (adjusted K-factor = 181) for 25 min (UC25min), 48 min (UC48min) or 60 min (UC60min), at 10 °C. Supernatant was discarded by decanting, SEVs pellet was drained and resuspended in 100 µL of PBS (Fig. 1a).

#### LEVs pelleting step testing: UCLEVs protocol

LEVs pelleting ultracentrifugation time was calculated by the Centrifugation Parameters Calculator, using the values described in Table 1^17,18,31^. The respective assumed LEVs density was 1.15 g/cm^3^^23^, which corresponds to the reported value of minimum density of LEVs. A 210 nm complete sedimentation cut-off size was used, to avoid pelleting of EVs with a mean size of 200 nm, as these are still considered “small EVs” (≤ 200 nm). Urine density and viscosity were considered the same as described above. With these parameters, 8 min of ultracentrifugation were calculated, plus the time ultracentrifuge requires until it reached 20,000*g* (2 min).

Considering the calculated time to pellet LEVs, urine supernatant was ultracentrifuged at 20,000*g* (adjusted K-factor = 831) for 10 min at 10 °C. The LEVs pellet was resuspended in 100 µL of PBS, and urine supernatant was ultracentrifuged at 200,000*g* for 48 min at 10 °C. The resulting supernatant was then discarded by decanting. Subsequently, SEVs pellet was drained and resuspended in 100 µL PBS (Fig. 1b).

#### Washing step testing of UC48min: UCwash protocol

Urine supernatant was filtered with a 0.22 µm pore membrane PVDF filter (Catalog number: SLGV033RB, Millipore, MA, USA) and ultracentrifuged at 200,000*g* during 48 min, at 10 °C. Subsequently, the supernatant was discarded by decanting, 1 mL of PBS was added to the SEVs pellet, which was resuspended by pipetting for 2 min. Afterward, 9 mL of PBS was added and briefly mixed by vortexing. Subsequently, a 200,000*g* ultracentrifugation during 48 min at 10°C was performed. Finally, the supernatant was removed by decanting, SEVs pellet was drained and resuspended in 100 µL of PBS (Fig. 1c).

### SEVs’ separation by precipitation (EXO)

SEVs were isolated from 10 mL of urine using Exoquick-TC® precipitation solution (System Biosciences, CA, USA) following manufacturers’ protocol. Briefly, a low-speed centrifugation at 3000*g* for 15 min at 4 °C was performed to clean urine sediments and cells. The resulting supernatant was decanted into 15 mL polypropylene tubes (Labbox, Barcelona, Spain) and 2 mL of Exoquick solution was added and incubated overnight at 4 °C. SEVs were further enriched by low-speed centrifugations as described in the manufacturers protocol (Cat# EXOTC10A-1 and Cat# EXOTC50A-1, Version 8, 2016).

### SEVs' separation by density ultracentrifugation (dUC)

The dUC procedure was adapted from the protocol of Maia et al.^32^. Briefly, urine supernatant was centrifuged at 500*g* for 10 min at 10 °C, to pellet the cells. Afterwards, supernatant was centrifuged at 3000*g* during 20 min at 10 °C, and the debris pellet was discarded. The collected supernatant was then ultracentrifuged at 12,000*g* for 20 min at 10 °C, and the LEV pellet was removed. Subsequently, samples were submitted to a 100,000*g* ultracentrifugation at 10 °C for 2 h 20 min, and the SEV pellet was resuspended in 16 mL of PBS. Subsequently, a 30% sucrose cushion was loaded into the tube and 16 mL of the previously diluted SEV pellet were added on top of the sucrose cushion. Then, a 1h 10 min 100,000*g* ultracentrifugation was performed at 10 °C, followed by the collection of the sucrose fraction which was subsequently diluted in 16 mL of PBS. Finally, a 16 h 100,000*g* ultracentrifugation was carried out at 10 °C, and SEV pellet was resuspended in 100 µL of PBS.

### EVs’ characterization

#### Nanoparticle tracking analysis (NTA)

Nanoparticle Tracking Analysis (NTA) was performed in a Nanosight NS300 system (Malvern Panalytical, Malvern, UK) equipped with a blue laser. EV volume was adjusted with PBS to a total volume of 1 mL (dilutions ranging 1:33–1:1000) and loaded in a 1 mL syringe. A continuous syringe pump flow of three was used, and target temperature was 25 °C for all experiments. Five videos of 30 s were recorded with a minimum of 1000 total tracks and a range of 10–50 particles per frame to ensure valid results. A screen gain of nine and a camera level of 10 were used in all captures. Videos were recorded and analyzed with NTA software 3.4 version to estimate particle number and size distribution.

#### Protein quantification

Protein concentration of EV samples from UC and dUC was quantified using the Micro BCA™ Protein Assay Kit (Thermo Fisher Scientific, MA, USA), following manufacturer’s instructions. Protein quantification of EV samples isolated using Exoquick was performed with Pierce™ BCA Protein Assay Kit (Thermo Fisher Scientific, MA, USA) (following manufacturers’ protocol) since EXO EVs contain high protein concentration, often out of the limit of quantification of Micro BCA™ Protein Assay Kit. Protein was quantified in a FLUOstar Omega microplate reader (BMG LABTEC, Ortenberg, German) at 562 nm.

#### Western-blot (WB)

EV markers (CD9, CD81, CD63, Flotillin-1 and Alix), the urine contaminant THP (Tamm-Horsfall Protein) and the intracellular components Cytochrome C and Lamin A/C were assessed by Western-blot (WB).

Equal volumes of EVs’ samples (20 µL) as described by Royo et al.^33^, were suspended in sample loading buffer (2-mecarptoethanol, glycerol, SDS, EDTA and bromophenol blue) and incubated at 95 °C for 5 min for EVs lysis and protein denaturation (means of particle number loaded per well are described in Supplementary Tables S2, S3, S4, S5, S6 and S8). Further, samples were separated by sodium dodecyl sulfate polyacrylamide gel electrophoresis (SDS-PAGE) using 4–20% mini-PROTEAN TGX Precast Gels (Bio-Rad Laboratories, CA, USA). After, the separated proteins were transferred to polyvinylidene difluoride (PVDF) membranes (Bio-Rad Laboratories, CA, USA) using a Trans-Blot®Turbo TM transfer system (Bio-Rad Laboratories, CA, USA). Membranes were then blocked using 1 × Tris-buffered saline (TBS) containing 5% bovine serum albumin (BSA) and 0.1% Tween20, for 1h30 min, at room temperature (RT). Subsequently, membranes were cut (before hybridization with antibodies) and incubated during 1h, at RT, with primary antibodies against CD9 (1:500; sc-13118, Santa Cruz Biotechnology, Texas, USA), CD81 (1:500; sc-166029, Santa Cruz Biotechnology, Texas, USA), CD63 (1:300; sc-5275, Santa Cruz Biotechnology), Flotillin-1 (1:300 sc-74566, Santa Cruz Biotechnology, Texas, USA), Alix (1:300; sc-53540, Santa Cruz Biotechnology, Texas, USA), THP (1:500; sc-271022, Santa Cruz Biotechnology, Texas, USA), Cytochrome C (1:500, sc-13560, Santa Cruz Biotechnology, Texas, USA) and Lamin A/C (1:500, sc-376248, Santa Cruz Biotechnology, Texas, USA) followed by incubation with a secondary antibody (1:5000; 7076S, Cell Signaling Technology, MA, USA) during 1h, at RT. Finally, membranes were exposed to Clarity WB ECL substrate (Bio-Rad Laboratories, CA, USA) and developed using X-ray films or ChemiDoc™ Imaging System (Bio-Rad Laboratories, CA, USA). Raw figures from Western-blots presented in this paper are depicted in Figs. S7–S11—Supplementary Material S2. Optical densitometry values were quantified using ImageJ 1.53K (National Institutes of Health, USA) and respective calculated values are in Tables S1–S6 from Supplementary Material S3.

#### Negative-staining transmission electron microscopy (TEM)

EV morphology was assessed by transmission electron microscopy (TEM), using negative staining. Firstly, 10 µL of EV samples were mounted on Formvar/carbon film-coated mesh nickel grids (Electron Microscopy Sciences, Hatfield, PA, USA) and left standing for 2 min. Subsequently, 10 µL of 1% uranyl acetate were added onto the grids and excess liquid was removed. Visualization was carried out on a JEOL JEM 1400 microscope (JEOL, Tokyo, Japan) at 120 kV. Images were recorded using a digital camera Orious 1100W (Tokyo, Japan).

#### RNA separation and analysis

RNA was isolated from 100 µL of SEVs samples using miRNeasy Serum/Plasma kit (Quiagen, Germany, Hilden) following manufacturer’s instructions. To evaluate RNase treatment effect, 0.2 µg/mL of RNase A (Sigma; R5503, Kanagawa, Japan) was added to SEV samples and incubated at 37 °C during 30 min before RNA extraction. Then, 1 U/µL of RNase A inhibitor (Thermo Fisher Scientific, MA, USA) was added and incubated at 37 °C for 30 min. RNA quantification and profiling was carried out with a Bioanalyzer 2100 Expert (Agilent Technologies, CA, USA) using Eukaryote Total RNA Pico assay (Agilent Technologies, CA, USA).

### EV-TRACK

All relevant data was submitted to the EV-TRACK knowledgebase (EV-TRACK ID: EV220193).

### Supplementary Information

Supplementary Information 1.

Supplementary Figures.

Supplementary Tables.

## Data Availability

All data generated or analysed during this study are included in this article.
