# Supplementary material for: Improved recovery of urinary small extracellular vesicles by differential ultracentrifugation
Source: Sci Rep. 2024 May 28;14:12267. doi: 10.1038/s41598-024-62783-9 (PMC11133306; doi:10.1038/s41598-024-62783-9)
Supplement: Supplementary file 1 — Supplementary Information 1. [file 41598_2024_62783_MOESM1_ESM.pdf]

Supplementary Material 1 - Teixeira-Marques *et al*. Improved recovery of urinary small extracellular vesicles by differential ultracentrifugation

Supplementary Table S1 - Clinicopathologic characteristics of the patients.

Supplementary Table S2 - Data corresponding to ultracentrifugation time period optimization (UC25min, UC48min and UC60min protocols).

Supplementary Table S3 - Information corresponding to large EVs (LEVs) pelleting (UCLEVs) versus urine supernatant filtering (UC48min).

Supplementary Table S4 - Information about characterization from SEVs isolated without washing step (UC48min) and with washing step (UCwash).

Supplementary Table S5 - Information of characterization from SEVs isolated with optimized differential ultracentrifugation (UC48min), density ultracentrifugation (dUC) and Exoquick (EXO).

Supplementary Table S6 - Information about characterization from SEVs isolated from 3 patients by UC48min and dUC methods.

Supplementary Table S7 - Information about RNA concentration obtained by UC48min and dUC method under RNAse and without RNAse conditions.

Supplementary Table S8 - Information about characterization from SEVs isolated from 7 patients.

Supplementary Figure S1 - Dot plots from particle concentration, particle mean size, protein concentration and purity ratio of all samples used among the tested conditions.

Supplementary Figure S2 - Western-blot characterization of small (SEVs) and large EVs (LEVs) pellet of LEVs pelleting (UCLEVs) and urine supernatant filtering (UC48min) comparisons.

Supplementary Figure S3 - Nanoparticle Tracking Analysis and Western-blot from SEVs separated with UC48min and dUC methods.

Supplementary Figure S4 - Nanoparticle Tracking Analysis from comparisons of UC48min protocol without treatment, with 2-mecarptoethanol treatment and NaCl treatment.

Supplementary Figure S5 - Bioanalyzer graphics representing RNA size distribution of UC48min and dUC in RNAse treated and non-treated samples. Comparisons in three independent urine samples are demonstrated.

Supplementary Figure S6 - Western-blot from 7 independent urine samples isolated with UC48min protocol.

Supplementary Figure S7 – Dot blots from SEV-RNA concentration obtained by Bioanalyzer analysis from sixteen independent patients. Horizontal red line corresponds to the mean of tested samples.

Supplementary Method S1 – UC48min with 2-Mecarptoethanol treatment

Supplementary Method S2 – UC48min with NaCl treatment

Supplementary tables

Supplementary Table S1. Clinicopathologic characteristics of the patients.

**Clinicopathologic features**	
Healthy donors	*9*
Cancer patients	*29*
Healthy donors	
Age [years (median, interquartile range)]	36.0 (46.0)
Age segregated by gender	*[years (median, interquartile range)]*
Male	32.5 (46.0)
Female	63.0 (0.0)
Gender	
Male	8/9 (72.0)
Female	1/9 (28.0)
Cancer patients	
Age [years (median, interquartile range)]	65.0 (40.0)
Age segregated by gender	*[years (median, interquartile range)]*
Male	64.5 (37.0)
Female	66.0 (31.0)
Gender	
Male	18/26 (69.2)
Female	8/26 (30.8)
Cancer type segregated by gender	
Male	
Bladder Cancer	9 (50.0)
Colorectal Cancer	2 (11.1)
Head and Neck Cancer	1 (5.5)
Prostate Cancer	4 (22.2)
Renal Cancer	1 (5.5)
Lung Cancer	1 (5.5)
Female	
Bladder Cancer	3 (33.3)
Breast Cancer	4 (50.0)
Colorectal Cancer	1 (12.5)

Supplementary Table S2. Data corresponding to ultracentrifugation time period optimization (UC25min, UC48min and UC60min protocols).

	Total particle recovery [Part]	Particle concentration [Part/mL]	Particle size mean [nm]	Total protein recovery [µg]	Protein concentration [µg/mL]	Purity ratio [Part/µg]	Particle number in 20 µL of EV sample
**UC25min**	1.56E+10 +/- 5.97E+08	1.56E+11 +/- 5.97E+09	168.3 +/- 3.9	74.71	747.1	2.19E+08	3.11E+09
**UC48min**	2.48E+10 +/- 9.22E+08	2.48E+11 +/- 9.22E+09	163.4 +/- 2.3	84.24	842.4	3.41E+08	4.95E+09
**UC60min**	1.90E+10+/- 5.38E+08	1.90E+11 +/- 5.38E+09	169 +/- 3.7	90.45	904.5	2.83E+08	3.39E+09

Total particle recovery, particle concentration particle size mean, and respective standard deviations measured by Nanoparticle Tracking Analysis are represented. Total protein recovery, protein concentration (microBCA) and purity ratio values (calculated by dividing particle concentration by protein concentration) are also described. Particle number in 20 µL of sample loaded for Western-Blot is represented. All measurements were based on 3 independent experimental replicates.

Supplementary Table S3. Information corresponding to large EVs (LEVs) pelleting (UCLEVs) versus urine supernatant filtering (UC48min).

	Total particle recovery [Part]	Particle concentration [Part/mL]	Particle size mean [nm]	Total protein recovery [µg]	Protein concentration [µg/mL]	Purity ratio [Part/µg]	Particle number in 20 µL of EV sample
**UC48min**	8.72E+10 +/- 3.26E+09	8.72E+11 +/- 3.26E+10	202.83 +/- 4.5	180.55	1805.5	4.38E+08	1.74E+10
**UCLEVs (SEVs)**	5.10E+10+/- 2.18E+09	5.10E+11 +/- 2.18E+10	209.8 +/- 6.0	169.31	1693.1	3.77E+08	1.02E+10
**UCLEVs (LEVs)**	1.91E+10+/- 1.14E+09	1.91E+11 +/- 1.14E+10	250.76 +/- 5.4	138.29	1382.9	1.59E+08	3.81E+09

Total particle recovery, particle concentration, particle size mean with respective standard deviations obtained by Nanoparticle Tracking Analysis. Total protein recovery and protein concentration measured using microBCA. Purity ratio and particle number in 20 µL of EV sample (loaded in Western-blot) are also represented. Values were based on 3 independent samples.

Supplementary Table S4. Information about characterization from SEVs isolated without washing step (UC48min) and with washing step (UCwash).

	Total particle recovery [Part]	Particle concentration [Part/mL]	Particle size mean [nm]	Total protein recovery [µg]	Protein concentration [µg/mL]	Purity ratio (Part/µg)	Particle number in 20 µL of EV sample
**UC48min**	2.48E+10 +/- 9.22E+08	2.48E+11 +/- 9.22E+09	163.4 +/- 2.2	84.24	842.4	1.09E+09	4.95E+09
**UCwash**	1.16E+10 +/- 5.26E+08	1.16E+11 +/- 5.26E+09	173.1 +/- 3.3	8.84	88.4	2.77E+09	2.32E+09

Values from total particle recovery, particle concentration, particle size mean and respective standard deviations measured using Nanoparticle Tracking Analysis. Total protein recovery and protein

concentration (assessed by microBCA), purity ratio and particle number in 20 µL of sample loaded in Western-Blot are represented. Values were based on 3 independent experimental replicates.

Supplementary Table S5. Information of characterization from SEVs isolated with optimized differential ultracentrifugation (UC48min), density ultracentrifugation (dUC) and Exoquick (EXO).

	Total particle recovery [Part]	Particle concentration [Part/mL]	Particle size mean [nm]	Total protein recovery [µg]	Protein concentration [µg/mL]	Purity ratio (Part/µg]	Particle number in 20 µL of EV sample
**UC48min**	4.46E+10 +/- 1.86E+09	4.46E+11 +/- 1.86E+10	159.6 +/- 3.7	87.98	879.8	5.81E+08	8.93E+09
**dUC**	2.34E+10 +/- 7.37E+09	2.34E+11 +/- 7.37E+09	159.6 +/- 3.3	10.18	101.8	2.67E+09	4.68E+09
**EXO**	4.33E+10 +/- 2.87E+09	4.33E+11 +/- 2.87E+10	174.43 +/- 2.6	135.41	1354.1	2.98E+08	8.67E+09

Values from total particle recovery, particle concentration, particle size mean with standard deviations from Nanoparticle Tracking Analysis. Total protein recovery, protein concentration (measured by microBCA), purity ratio and particle number in 20 µL of EV sample used for Western-Blot are represented. Values were based on 3 independent samples.

Supplementary Table S6. Information about characterization from SEVs isolated from 3 patients by UC48min and dUC methods.

	Total particle recovery [Part]	Particle concentration [Part/mL]	Particle size mean [nm]	Total protein recovery [µg]	Protein concentration [µg/mL]	Purity ratio [Part/µg]	Particle number in 20 µL of EV sample
**UC48min**	3.85E+10 +/- 1.84E+09	3.85E+11 +/- 1.84E+10	169.5 +/- 4.0	178.7	1787.3	2.80E+08	2.31E+10
**dUC**	6.07E+09 +/- 2.69E+08	6.07E+10 +/- 2.69E+09	160.1 +/- 4.1	2.7	26.6	2.28E+09	3.64E+09

Average values from total particle recovery, particle concentration, particle size mean and standard deviations from Nanoparticle Tracking Analysis. Average values from total protein recovery and protein concentration (evaluated using microBCA), purity ratio and particle number in 20 µL of EV sample loaded for Western-Blot are represented. Values are based on 3 independent sample replicates.

Supplementary Table S7. Information about RNA concentration obtained by UC48min and dUC method under RNAse and without RNAse conditions.

	With RNAse treatment [pg/µL]	Without RNAse treatment [pg/µL]
**UC48min**	123.3	146.0
**dUC**	43.0	127.0

Supplementary Table S8. Information about characterization from SEVs isolated from 7 patients.

	Total particle recovery [Part]	Particle concentration [Part/mL]	Particle size mean [nm]	Total protein recovery [µg]	Protein concentration [µg/mL]	Purity ratio (Part/µg]	Particle number in 20 µL of EV sample
**UC48min**	2.55E+10 +/- 9.30E+08	2.55E+11 +/- 9.30E+09	213.4 +/- 4.5	122.6	1226.0	4.47E+08	5.09E+09

Average values from total particle recovery, particle concentration, particle size mean and standard deviations from Nanoparticle Tracking Analysis. Average values from total protein recovery and protein concentration (evaluated using microBCA), purity ratio and particle number in 20 µL of EV sample loaded for Western-Blot are represented values based on 7 independent sample replicates.

Supplementary Figures

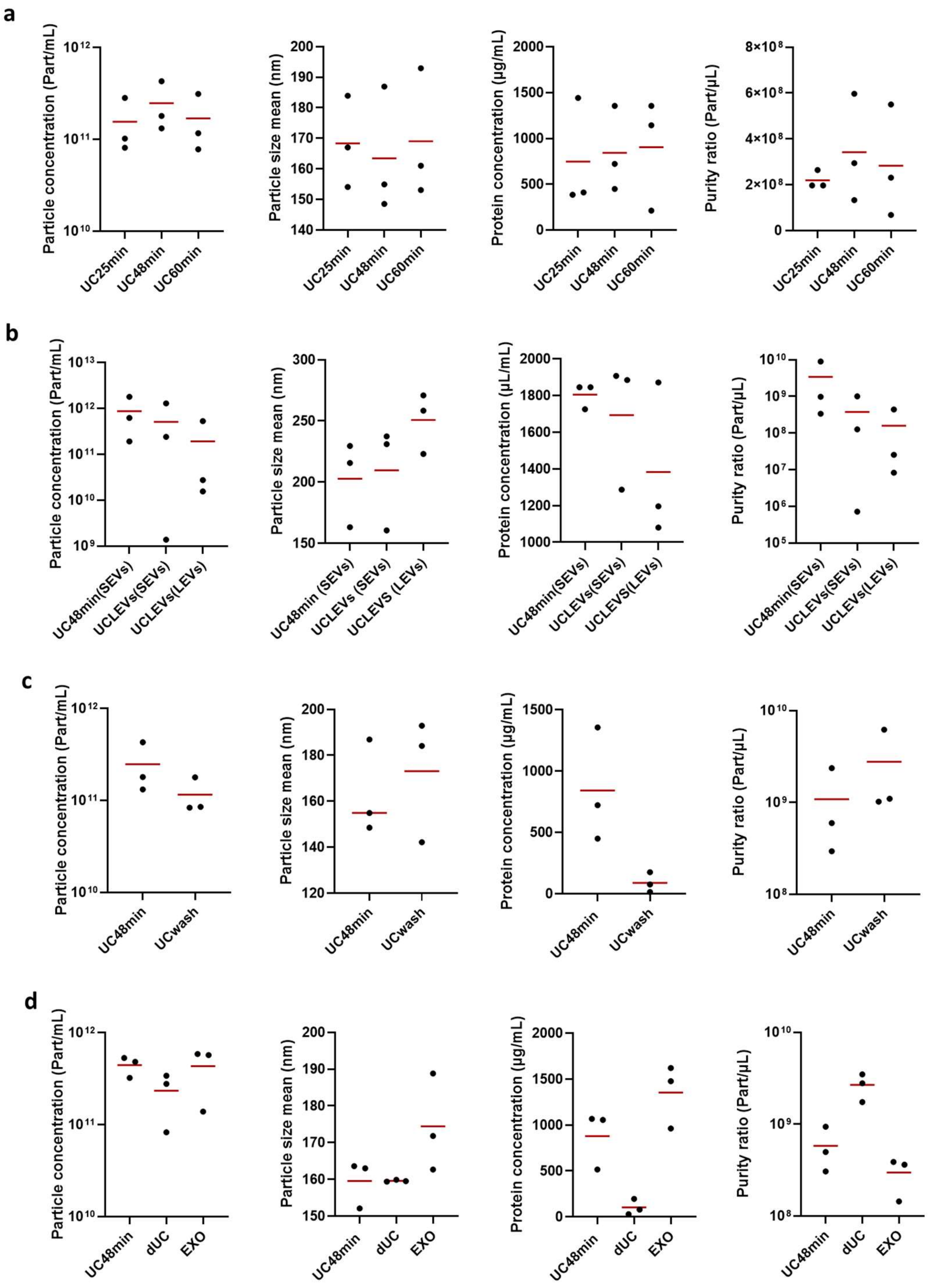

Supplementary Figure S1. Dot plots from particle concentration, particle mean size, protein concentration and purity ratio of all samples used among the tested conditions. **(a)** Protocols including 25 min (UC25min), 48 min (UC48min) and 60 min (UC60min) ultracentrifugation times. **(b)** Comparison of small (SEVs) and large (LEVs) EVs pelleting (UCLEVs) with urine supernatant filtering (UC48min). **(c)** Protocols with (UCwash) and without (UC48min) washing step. **(d)** Comparison between optimized differential ultracentrifugation (UC48min), density ultracentrifugation (dUC) and Exoquick (EXO). Horizontal red lines represent the means of tested samples per condition. All parameters were measured in 3 samples for each tested condition.

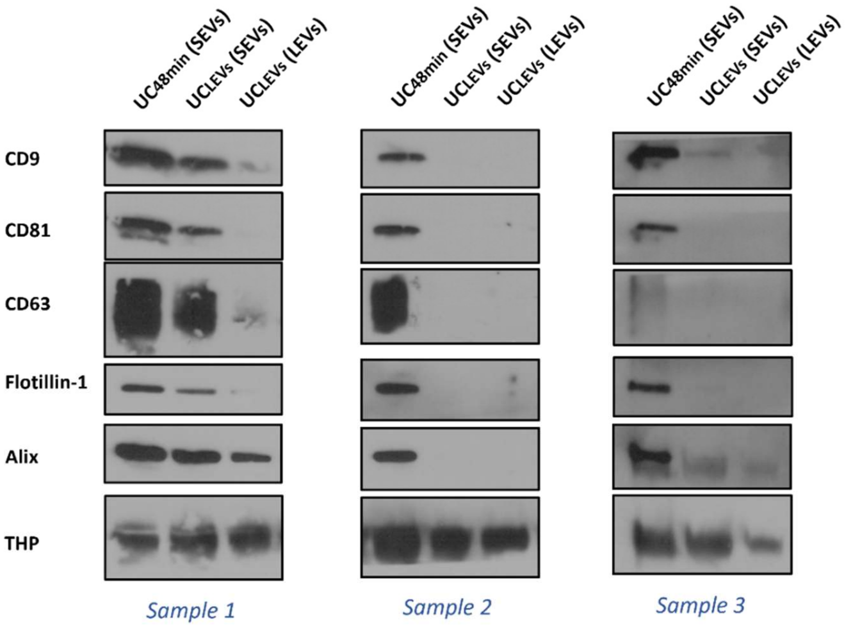

Supplementary Figure S2. Western-blot characterization of small (SEVs) and large EVs (LEVs) pellet of LEVs pelleting (UCLEVs) and urine supernatant filtering (UC48min) comparisons. Western-blot for EV-classical markers CD9, CD81, CD63, Flotillin-1 and Alix and THP of 3 independent sample replicates are represented. Western-blot images were cropped; the original blots are presented in Figure S8 from Supplementary Material 2.

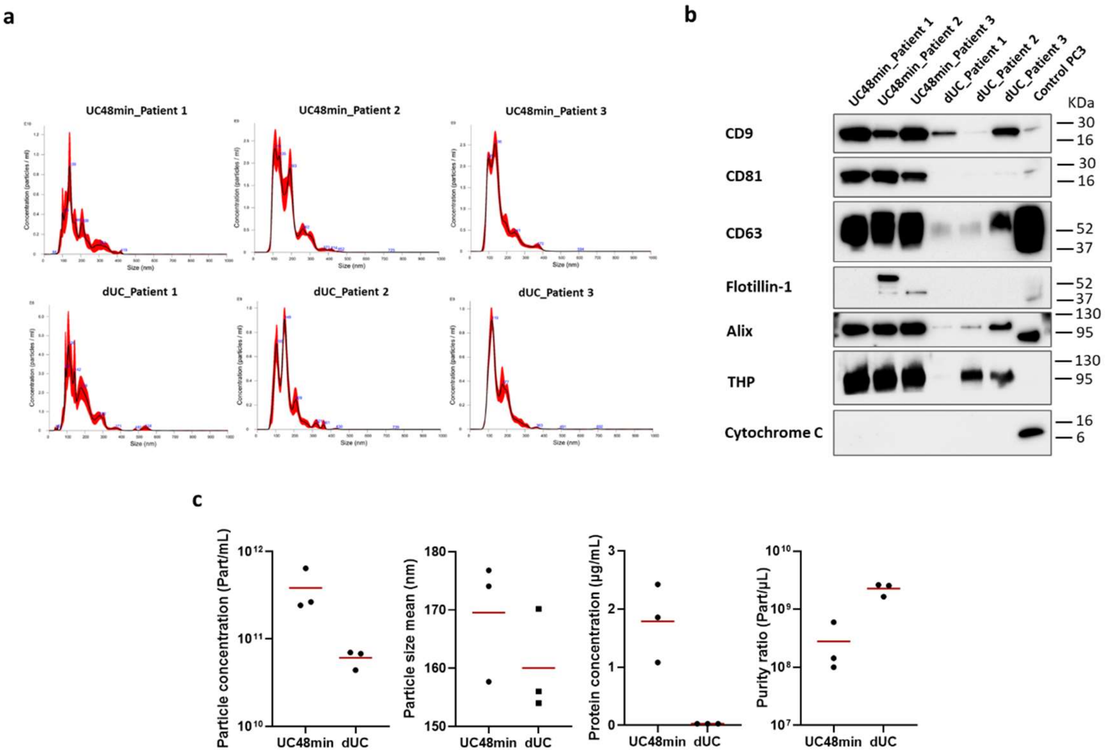

Supplementary Figure S3. Nanoparticle Tracking Analysis and Western-blot analysis of SEVs separated with UC48min and dUC methods. **(a)** Nanoparticle tracking analysis (NTA) graphs display in y-axis: concentration (Particle/mL), and in x-axis: size (nm). **(b)** EV-classical markers CD9, CD81, CD63, Flotillin-1

and Alix are represented in different samples. THP (urine contaminant), Lamin A/C and Cytochrome C (associated with intracellular compartments) markers are also represented. PC3 cell line lysate was used as a positive control for Lamin A/C and Cytochrome C markers**.** Western-blot images were cropped; the original blots are presented in Figure S10 from Supplementary Material 2. **(c)** Dot plots of particle concentration, particle mean size, protein concentration and purity ratio results for all samples used among the tested conditions. Horizontal black lines represent the means of tested samples per condition. All parameters were measured in 3 samples for each tested method.

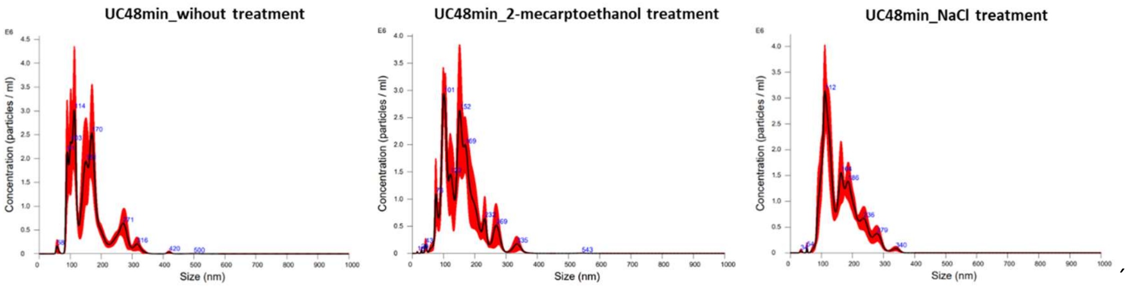

Supplementary Figure S4. Graphical representations of Nanoparticle Tracking Analysis regarding the comparisons of UC48min protocol without any treatment, with 2-mecarptoethanol treatment and with NaCl treatment. Nanoparticle tracking analysis (NTA) graphs display in y-axis: concentration (Particle/mL), and in x-axis: size (nm). Experiments were conducted in 3 independent patients.

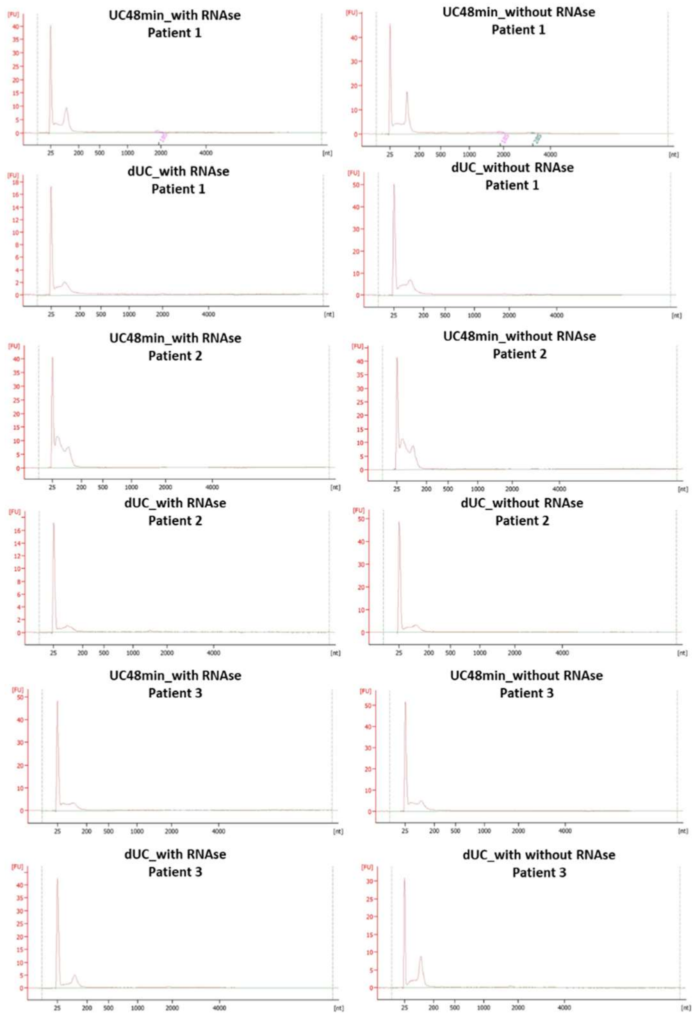

Supplementary Figure S5. Bioanalyzer graphics representing RNA size distribution of UC48min and dUC in RNAse treated and non-treated samples. Comparisons in three independent urine samples are demonstrated.

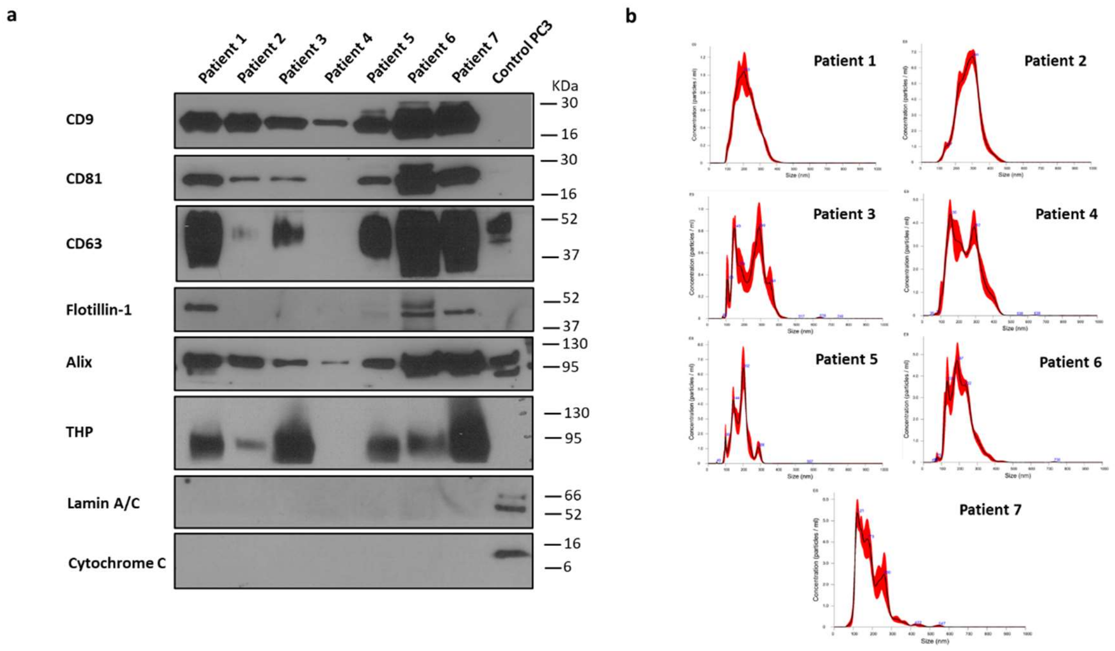

Supplementary Figure S6. Western-blot and Nanoparticle Tracking Analysis from 7 independent urine samples isolated with UC48min protocol. **(a)** EV-classical markers CD9, CD81, CD63, Flotillin-1 and Alix are represented in different samples. THP (urine contaminant), Lamin A/C and Cytochrome C (associated with intracellular compartments) markers are also represented. PC3 cell line lysate was used as a positive control for Lamin A/C and Cytochrome C markers. Western-blot images were cropped; the original blots are presented in Figure S11 from Supplementary Material 2. **(b)** Nanoparticle tracking analysis (NTA) graphs display in y-axis: concentration (Particle/mL), and in x-axis: size (nm).

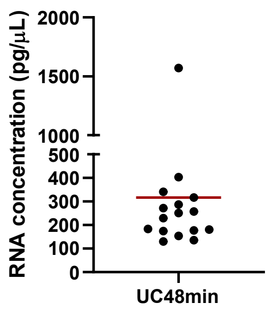

Supplementary Figure S7. Dot blot representing SEV-RNA concentration values obtained by Bioanalyzer analysis from sixteen independent patients. Horizontal red line corresponds to the mean of tested samples.

Supplementary Methods

Supplementary Method S1 – UC48min with 2-Mecarptoethanol treatment

Voided urine was collected and centrifuged at 3700g (in a Gyrozen 1580R centrifuge; Gyrozen, St Petersburg, Russia) during 20 min, at 4 °C to pellet cells and debris. The resultant supernatant was decanted and stored in 50 mL polypropylene tubes (Nerbe plus; Winsen, Germany) at -80 °C until further use.

After thawing, 10 mL urine supernatant from each patient was vortexed and a centrifugation at 3,000 g during 15 min at 4 °C in a Sigma 3-16PK centrifuge (Sigma, Missouri, USA) was performed. Urine supernatant was ultracentrifuged at 20,000g (adjusted K-factor=831) for 10 min at 10 °C. The supernatant was transferred to a separated tube, while LEVs pellet was resuspended in 975 µL of 50mM Tris and 25 µL of 2-mecarptoethanol and vortexed, proceeded by an incubation for 30 min at room temperature (this procedure was adapted from Santucci et al 2019 [1]). Afterwards, LEVs pellet was mixed with the supernatant, filtered using a 0.22 µm PVDF filter and ultracentrifuged at 200,000g for 48 min at 10°C. The resulting supernatant was then discarded by decanting. Subsequently, SEVs pellet was drained and resuspended in 100 µL PBS.

Supplementary Method S2 – UC48min with NaCl treatment

Voided urine was collected and centrifuged at 3700 g (in a Gyrozen 1580R centrifuge; Gyrozen, St Petersburg, Russia) during 20 min, at 4 °C to pellet cells and debris. The resultant supernatant was decanted and stored in 50 mL polypropylene tubes (Nerbe plus; Winsen, Germany) at -80 °C until further use.

After thawing 10 mL urine supernatant from each patient, a 0.58 mM NaCl solution was added to each sample and the mixture was vortexed and incubated for 2h at room temperature (procedure adapted from Kosanović *et al* 2014 [2]). Afterwards, the mixture was vortexed to disperse EVs and protein aggregates following by a centrifugation at 3000g during 15 min at 4 °C in a Sigma 3-16PK centrifuge (Sigma, Missouri, USA) to pellet and discard urine sediment remains. The supernatant was filtered using a 0.22 µm PVDF filter and ultracentrifuged at 200,000g for 48 min at 10°C. The resulting supernatant was then discarded by decanting. Subsequently, SEVs pellet was drained and resuspended in 100 µL PBS.

References

1 Santucci, L. *et al.* Biological surface properties in extracellular vesicles and their effect on cargo proteins. *Sci Rep* **9**, 13048 (2019). https://doi.org:10.1038/s41598-019-47598-3

2 Kosanović, M. & Janković, M. Isolation of urinary extracellular vesicles from Tamm-Horsfall protein–depleted urine and their application in the development of a lectin-exosome-binding assay. *BioTechniques* **57**, 143-149 (2014). https://doi.org:10.2144/000114208
