# Supplementary material for: Improved recovery of urinary small extracellular vesicles by differential ultracentrifugation
Source: Sci Rep. 2024 May 28;14:12267. doi: 10.1038/s41598-024-62783-9 (PMC11133306; doi:10.1038/s41598-024-62783-9)
Supplement: Supplementary file 2 — Supplementary Figures. [file 41598_2024_62783_MOESM2_ESM.pdf]

Supplementary Material 2 – Teixeira-Marques *et al*. Improved recovery of urinary small extracellular vesicles by differential ultracentrifugation

Supplementary Figure S1 – Dot plots from optical densitometry values measured from CD9, CD81, CD63, Alix, Flotillin-1 and THP signals obtained by Western-blot of all samples used for UC25min, UC48min and UC60min protocols.

Supplementary Figure S2 – Dot plots from optical densitometry values measured from CD9, CD81, CD63, Alix, Flotillin-1 and THP signals obtained by Western-blot of all samples used for UC48min and UCLEVs protocols.

Supplementary Figure S3 – Dot plots of optical densitometry values from CD9, CD81, CD63, Alix, Flotillin-1 and THP signals obtained by Western-blot of all samples used for UC48min and UCwash protocols.

Supplementary Figure S4 – Dot plots from optical densitometry values measured from CD9, CD81, CD63, Alix, Flotillin-1 and THP signals obtained by Western-blot of all samples used for UC48min, dUC and EXO methods.

Supplementary Figure S5 – Dot plots from optical densitometry values measured from CD9, CD81, CD63, Alix, Flotillin-1, THP and Cytochrome C signals obtained by Western-blot of all samples used for UC48min, dUC and methods.

Supplementary Figure S6 – Dot plots from optical densitometry values measured from CD9, CD81, CD63, Alix, Flotillin-1, THP, Lamin A/C and Cytochrome C signals obtained by Western-blot of all samples used for UC48min, dUC and EXO methods. Horizontal black lines represent the means of tested samples per condition.

Supplementary Figure S7 – Raw figures corresponding to the Western-Blot from ultracentrifugation time period optimization (UC25min, UC48min and UC60min protocols) and UC48min versus UC with washing (UCwash).

Supplementary Figure S8 – Raw figure of the Western-blot corresponding to large EVs (LEVs) pelleting (UCLEVs) versus urine supernatant filtering (UC48min) protocols.

Supplementary Figure S9 – Raw figure of the Western-blot from optimized differential ultracentrifugation (UC48min), density ultracentrifugation (dUC) and Exoquick (EXO) methods.

Supplementary Figure S10 – Raw figures of the Western-blot from 3 independent urine EV samples separated using UC48min and dUC represented in Figure S3 from Supplementary Material 1.

Supplementary Figure S11 – Raw figure of the Western-blot from 7 independent urine samples separated using UC48min, represented in Figure S6 from Supplementary Material 1.

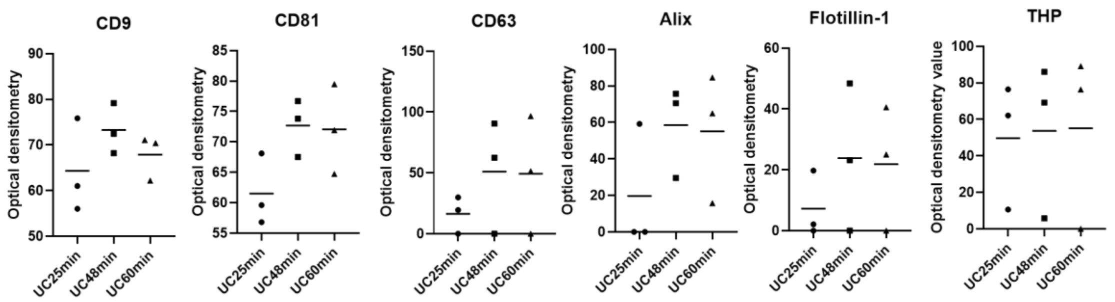

Supplementary Figure S1. Dot plots from optical densitometry values measured from CD9, CD81, CD63, Alix, Flotillin-1 and THP signals obtained by Western-blot of all samples used for UC25min, UC48min and UC60min protocols. Horizontal black lines represent the means of tested samples per condition.

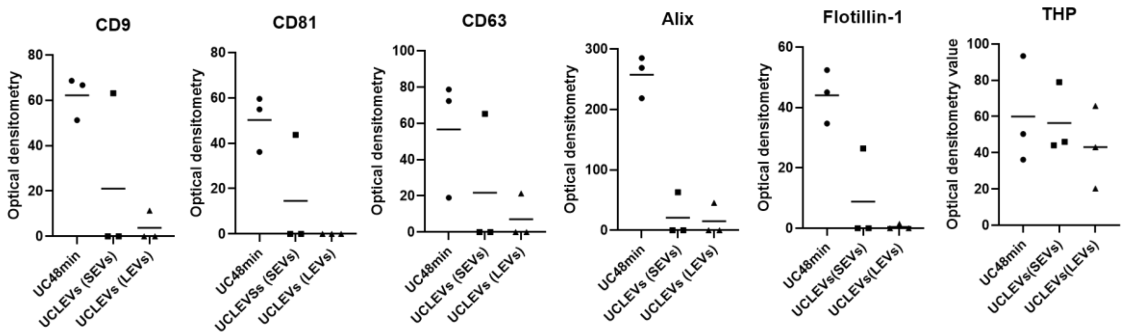

Supplementary Figure S2. Dot plots from optical densitometry values measured from CD9, CD81, CD63, Alix, Flotillin-1 and THP signals obtained by Western-blot of all samples used for UC48min and UCLEVs protocols. Horizontal black lines represent the means of tested samples per condition.

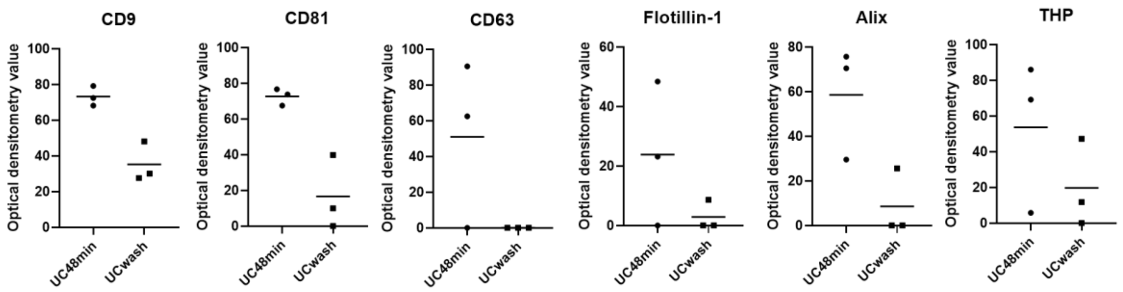

Supplementary Figure S3. Dot plots from optical densitometry values measured from CD9, CD81, CD63, Alix, Flotillin-1 and THP signals obtained by Western-blot of all samples used for UC48min and UCwash protocols. Horizontal black lines represent the means of tested samples per condition.

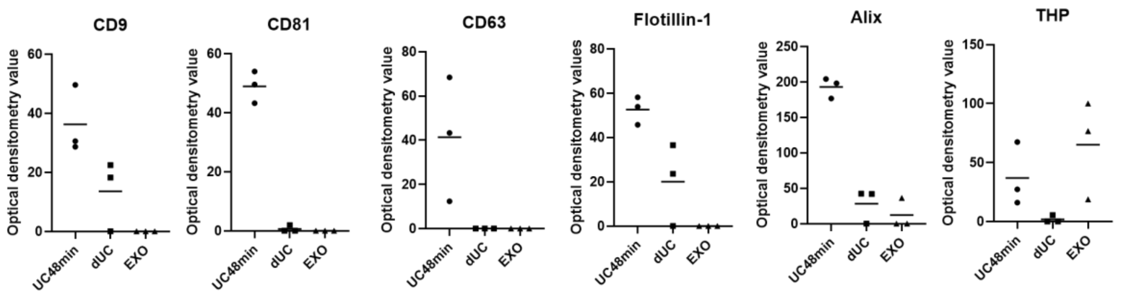

Supplementary Figure S4. Dot plots from optical densitometry values measured from CD9, CD81, CD63, Alix, Flotillin-1 and THP signals obtained by Western-blot of all samples used for UC48min, dUC and EXO methods. Horizontal black lines represent the means of tested samples per condition.

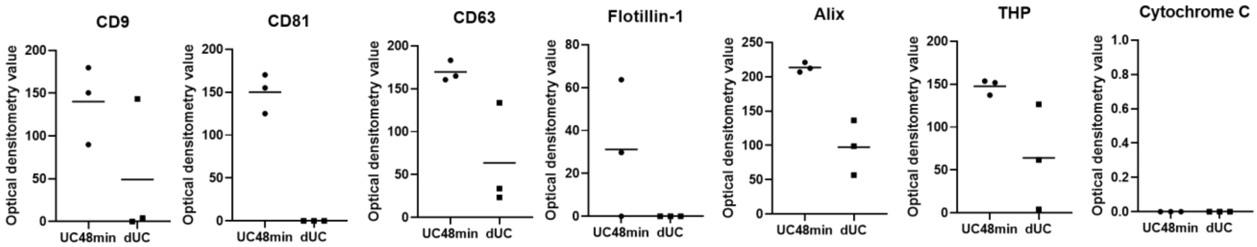

Supplementary Figure S5. Dot plots from optical densitometry values measured from CD9, CD81, CD63, Alix, Flotillin-1, THP and Cytochrome C signals obtained by Western-blot of all samples used for UC48min, dUC methods. Horizontal black lines represent the means of tested samples per condition.

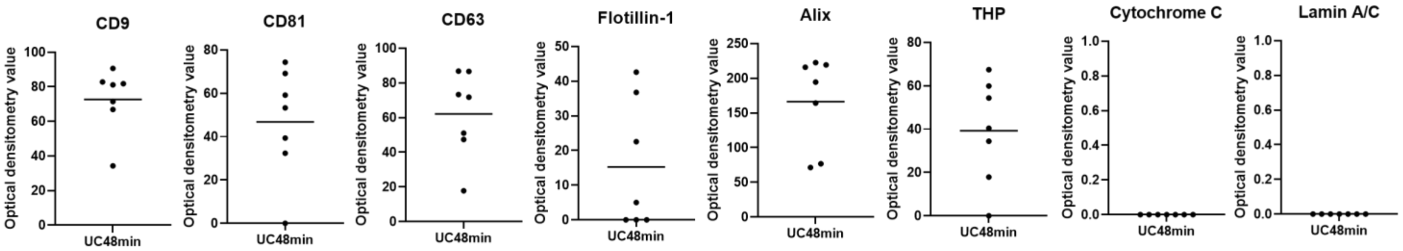

Supplementary Figure S6. Dot plots from optical densitometry values measured from CD9, CD81, CD63, Alix, Flotillin-1, THP, Lamin A/C and Cytochrome C signals obtained by Western-blot of all samples used for UC48min, dUC and EXO methods. Horizontal black lines represent the means of tested samples per condition.

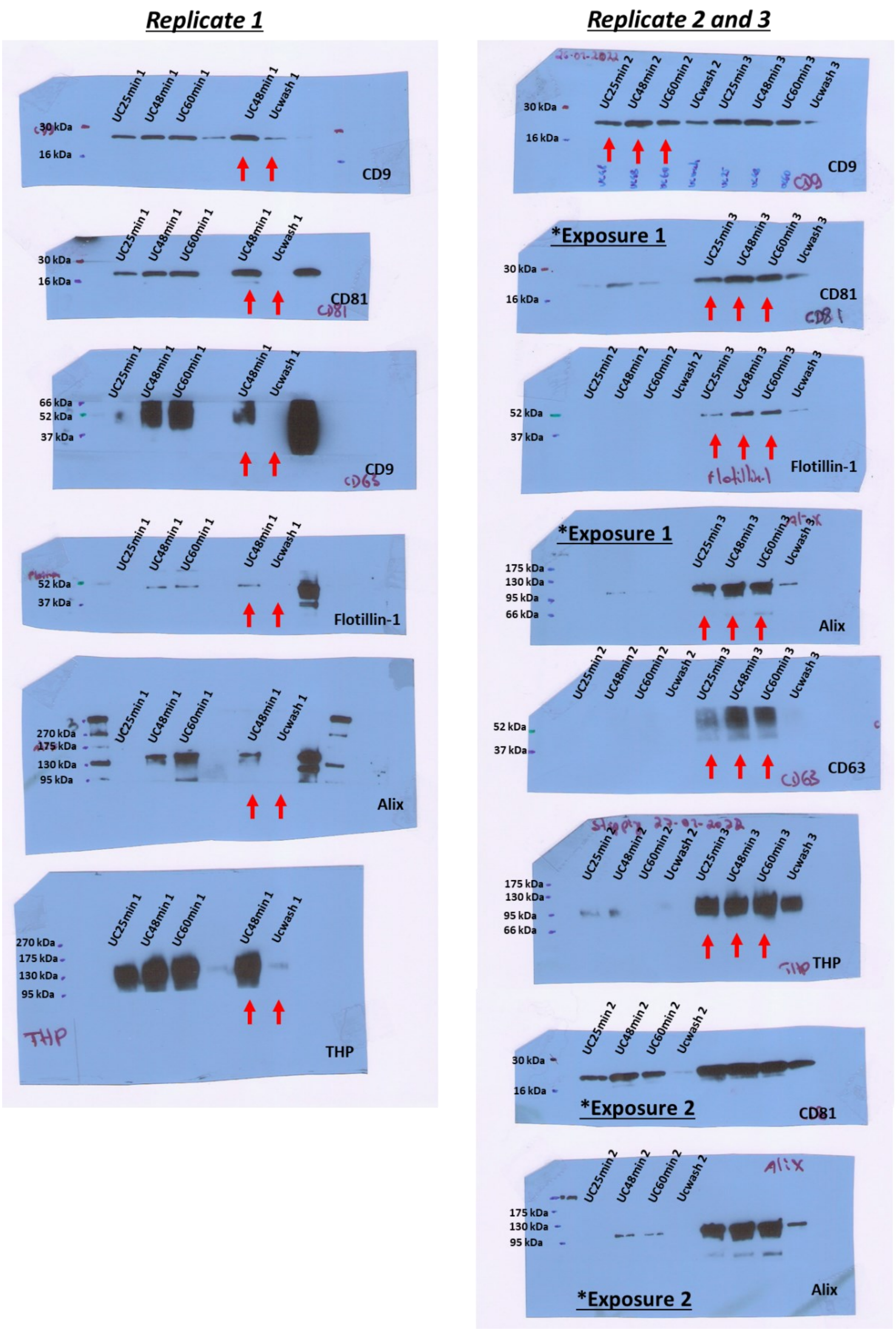

Supplementary Figure S7 – Raw figures corresponding to the Western-Blot from ultracentrifugation time period optimization (UC25min, UC48min and UC60min protocols) and UC48min versus differential ultracentrifugation with washing (UCwash). Red arrows pinpoint blots that were represented in Figure 2 and Figure 4. For each experiment 3 replicates were performed (replicates are referred as 1, 2 or 3). Films containing asterisks correspond to 2 different time exposures for CD81 and Alix in order to have the optimal signal for replicate 2 and 3. Optimal exposure for CD81 and Alix in replicate 2 was Exposure 2, while in replicate 3 for CD81 and Alix optimal exposure was Exposure 1.

Replicate 1 ***Replicate 2*** ***Replicate 3***

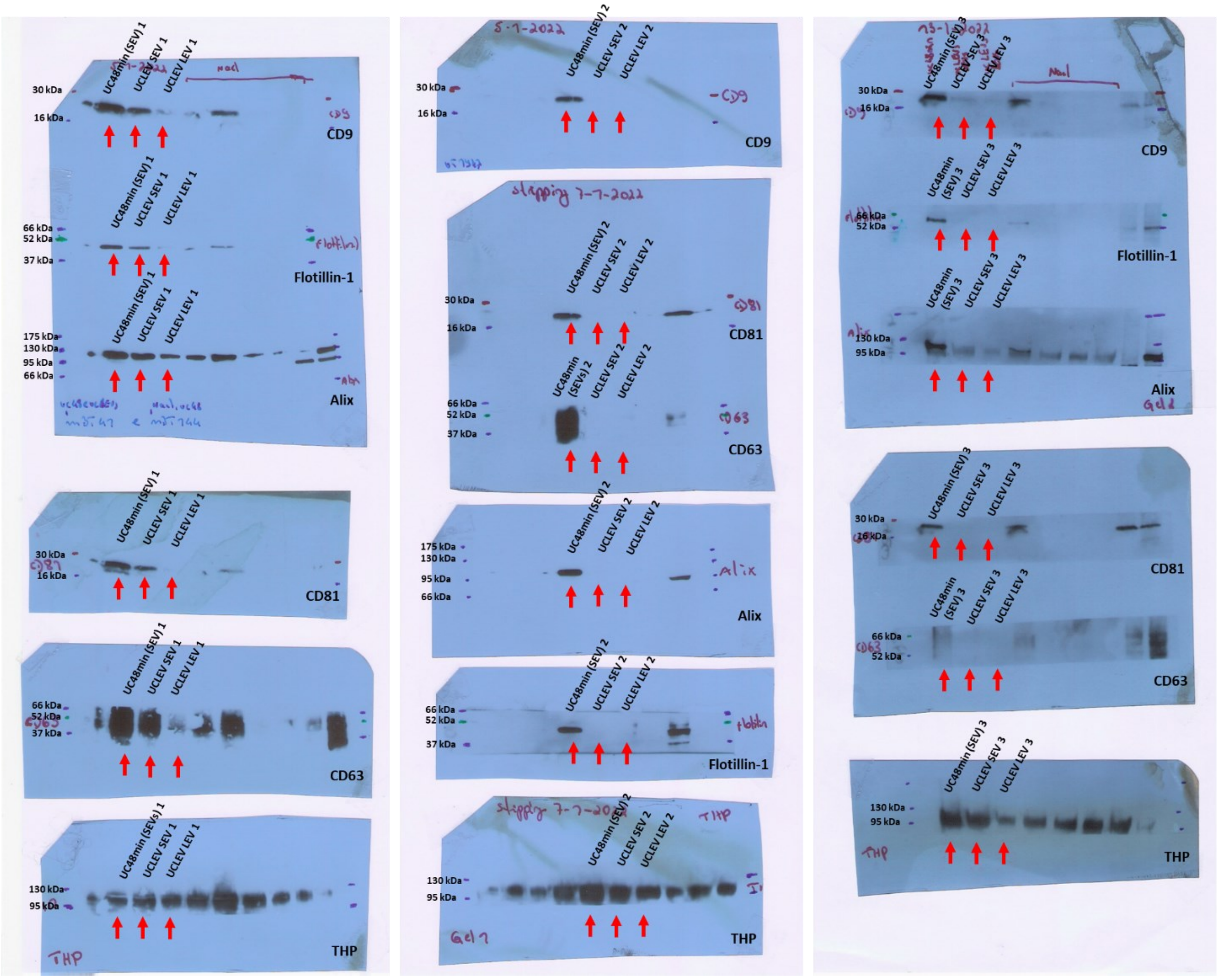

Supplementary Figure S8 – Raw figure of the Western-blot corresponding to large EVs (LEVs) pelleting (UCLEVs) versus urine supernatant filtering (UC48min) protocols. Red arrows pinpoint blots that ARE represented in Figure 3 and Figure S2 from Supplementary Material 1 (3 replicates were performed; replicates are referred as 1, 2 or 3).

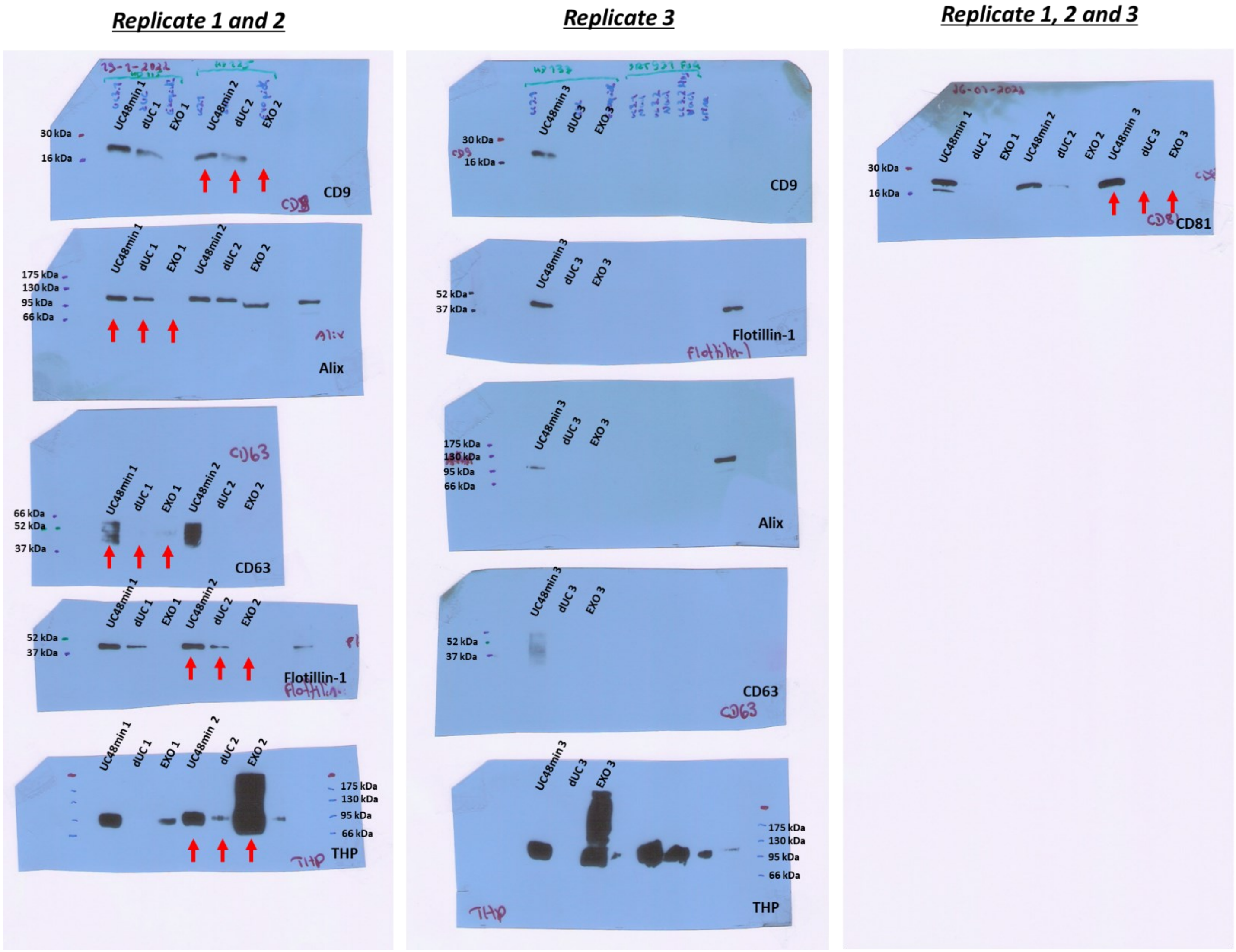

Supplementary Figure S9 – Raw figure of the Western-blot from optimized differential ultracentrifugation (UC48min), density ultracentrifugation (dUC) and Exoquick (EXO) methods. Red arrows pinpoint blots that were represented in Figure 5. For each experiment 3 replicates were performed (replicates are referred as 1, 2 or 3).

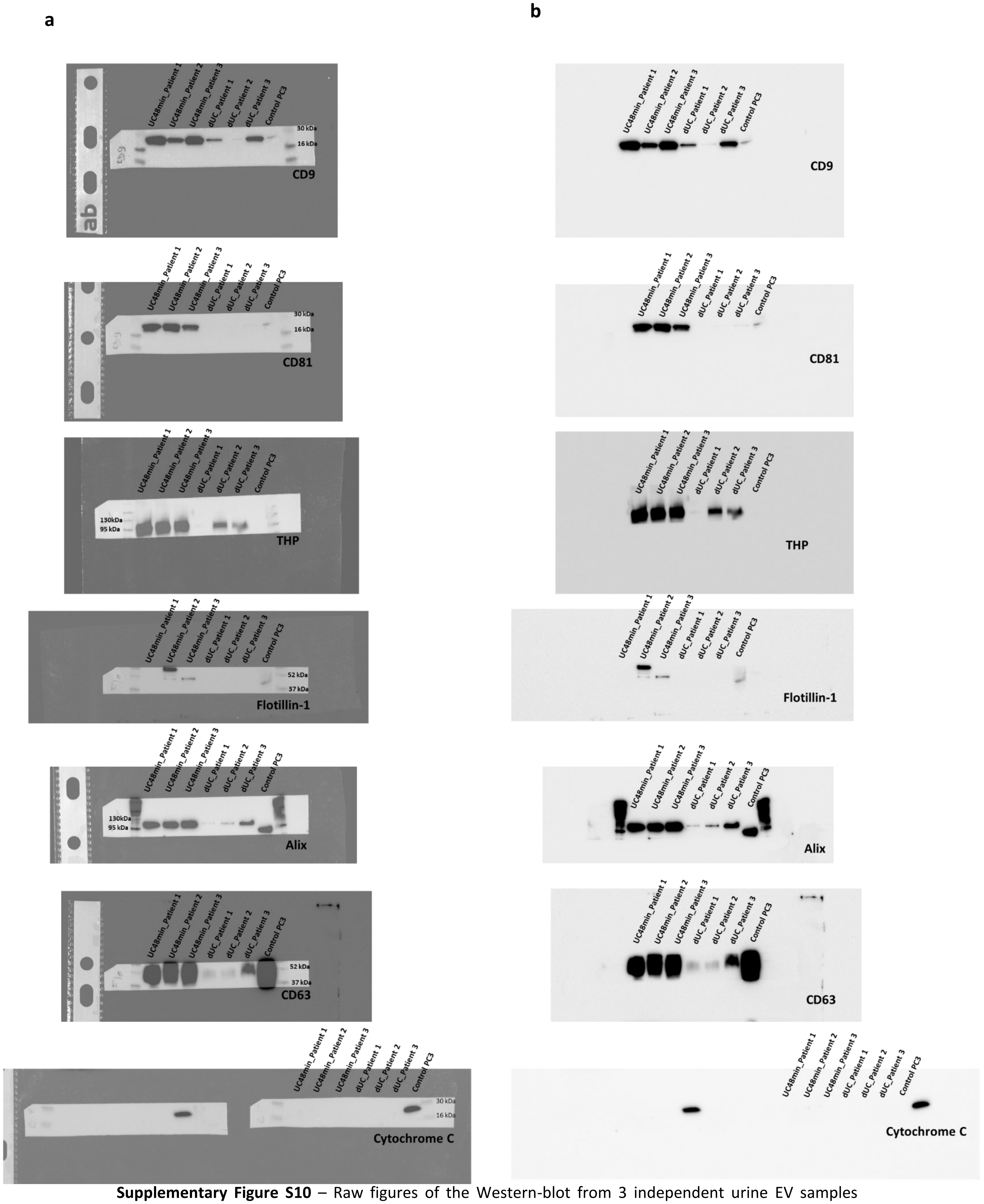

Supplementary Figure S10 – Raw figures of the Western-blot from 3 independent urine EV samples separated using UC48min and dUC that are represented in Figure S3 from Supplementary Material 1. **(a)** Raw image from Western-blot signal image merged with membrane image. **(b)** Raw image from Western-blot signal.

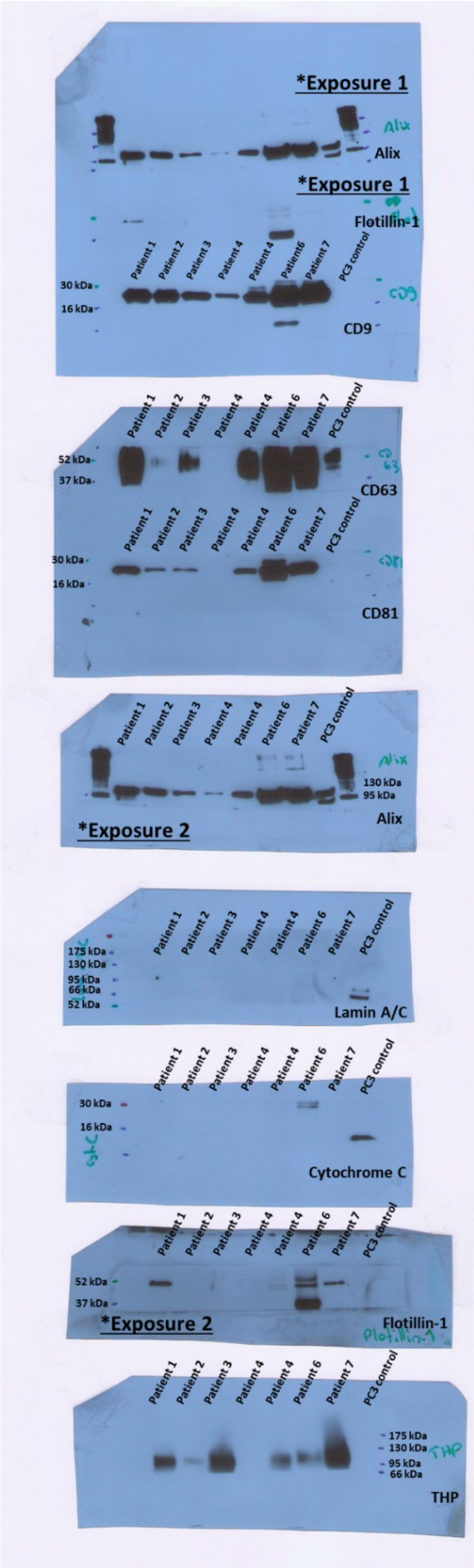

Supplementary Figure S11 – Raw image of the Western-blot from 7 independent urine samples separated using UC48min, represented in Figure S6 from Supplementary Material 1. Membranes containing asterisks are representative of 2 different time exposures for Alix and Flotillin-1. Exposure 2 was considered the optimal exposure for both Alix and Flotillin-1.
