# Supplementary material for: Improved recovery of urinary small extracellular vesicles by differential ultracentrifugation
Source: Sci Rep. 2024 May 28;14:12267. doi: 10.1038/s41598-024-62783-9 (PMC11133306; doi:10.1038/s41598-024-62783-9)
Supplement: Supplementary file 3 — Supplementary Tables. [file 41598_2024_62783_MOESM3_ESM.pdf]

Supplementary Material 3 - Teixeira-Marques *et al*. Improved recovery of urinary small extracellular vesicles by differential ultracentrifugation

Supplementary Table S1 – Calculated optical densitometry values of Western-blots correspondent to UC25min, UC48min and UC60min protocols.

Supplementary Table S2 – Calculated optical densitometry values of Western-blots correspondent to UC48min and UCLEVs protocols.

Supplementary Table S3 – Calculated optical densitometry values of Western-blots correspondent to UC48min and UCwash protocols.

Supplementary Table S4 – Calculated optical densitometry values of Western-blots correspondent to UC48min and dUC and EXO protocols.

Supplementary Table S5 – Calculated optical densitometry values of Western-blots correspondent to UC48min and dUC methods.

Supplementary Table S6 – Calculated optical densitometry values of Western-blots correspondent to UC48min protocol.

Supplementary Table S1. Calculated optical densitometry values of Western-blots correspondent to UC25min, UC48min and UC60min protocols.

Figure 2		CD9					CD81					CD63				
		CD9	inverted	background	inverted	OD	CD81	inverted	background	inverted	OD	CD63	inverted	background	inverted	OD
UC25min	1	119,804	135,20	180,833	74,17	**61,03**	117,608	137,392	177,216	77,784	**59,608**	159,973	95,027	179,536	75,464	19,563
	2	129,23	125,77	185,257	69,74	**56,03**	111,965	143,035	180,068	74,932	**68,103**	181,112	73,888	181,112	73,888	0
	3	109,412	145,59	185,257	69,74	**75,85**	121,358	133,642	178,163	76,837	**56,805**	151,19	103,81	181,112	73,888	29,922
	Mean (n=3)					**64,30**					**61,50533333**					**16,495**
UC48min	1	112,615	142,39	180,833	74,17	**68,22**	109,668	145,332	177,216	77,784	**67,548**	89,02	165,98	179,536	75,464	90,516
	2	112,795	142,21	185,257	69,74	**72,46**	106,252	148,748	180,068	74,932	**73,816**	181,112	73,888	181,112	73,888	0
	3	106,077	148,92	185,257	69,74	**79,18**	101,457	153,543	178,163	76,837	**76,706**	118,611	136,389	181,112	73,888	62,501
	Mean (n=3)					**73,29**					**72,69**					**51,00566667**
UC60min	1	110,431	144,57	180,833	74,17	**70,40**	97,722	157,278	177,216	77,784	**79,494**	82,826	172,174	179,536	75,464	96,71
	2	123,09	131,91	185,257	69,74	**62,17**	115,322	139,678	180,068	74,932	**64,746**	181,112	73,888	181,112	73,888	0
	3	114,172	140,83	185,257	69,74	**71,09**	106,23	148,77	178,163	76,837	**71,933**	129,707	125,293	181,112	73,888	51,405
	Mean (n=3)					**67,88**					**72,05766667**					**49,37166667**

Figure 2		Alix					Flotillin-1					THP				
		Alix	inverted	background	inverted	OD	Flottilin-1	inverted	background	inverted	OD	THP	inverted	background	inverted	OD
UC25min	1	174,536	80,464	174,536	80,464	0	176,574	78,426	178,674	76,326	2,1	99,799	155,201	176,295	78,705	76,496
	2	178,818	76,182	178,818	76,182	0	179,49	75,51	179,49	75,51	0	167,223	87,777	177,814	77,186	10,591
	3	119,419	135,581	178,6	76,4	59,181	159,733	95,267	179,49	75,51	19,757	115,654	139,346	177,814	77,186	62,16
	Mean (n=3)					**19,727**					**7,285666667**					**49,749**
UC48min	1	104,035	150,965	174,536	80,464	70,501	155,498	99,502	178,674	76,326	23,176	90,227	164,773	176,295	78,705	86,068
	2	149,26	105,74	178,818	76,182	29,558	179,49	75,51	179,49	75,51	0	172,038	82,962	177,814	77,186	5,776
	3	102,917	152,083	178,6	76,4	75,683	131,081	123,919	179,49	75,51	48,409	108,565	146,435	177,814	77,186	69,249
	Mean (n=3)					**58,58066667**					**23,86166667**					**53,69766667**
UC60min	1	89,932	165,068	174,536	80,464	84,604	153,593	101,407	178,674	76,326	25,081	87,179	167,821	176,295	78,705	89,116
	2	163,022	91,978	178,818	76,182	15,796	179,49	75,51	179,49	75,51	0	177,814	77,186	177,814	77,186	0
	3	113,602	141,398	178,6	76,4	64,998	138,924	116,076	179,49	75,51	40,566	101,471	153,529	177,814	77,186	76,343
	Mean (n=3)					**55,13266667**					**21,88233333**					**55,153**

Abbreviation: OD – Optical Densitometry value

Supplementary Table S2. Calculated optical densitometry values of Western-blots correspondent to UC48min and UCLEVs protocols.

Figure 3; Figure S2 in Supplementary Material 1		CD9					CD81					CD63				
		CD9	inverted	background	inverted	OD	CD81	inverted	background	inverted	OD	CD63	inverted	background	inverted	OD
UC48min	1	113,328	141,67	179,998	75,00	**66,67**	122,493	132,507	177,459	77,541	**54,966**	98,054	156,946	176,691	78,309	**78,637**
	2	83,864	171,14	152,51	102,49	**68,65**	104,56	150,44	140,728	114,272	**36,168**	130,786	124,214	149,711	105,289	**18,925**
	3	129,063	125,94	180,261	74,74	**51,20**	117,657	137,343	177,205	77,795	**59,548**	102,83	152,17	175,089	79,911	**72,259**
	Mean (n=3)					**62,17**					**50,22733333**					**56,607**
UCLEVS (SEVs)	1	116,886	138,11	179,998	75,00	**63,11**	133,728	121,272	177,459	77,541	**43,731**	111,487	143,513	176,691	78,309	**65,204**
	2	152,51	102,49	152,51	102,49	**0,00**	140,728	114,272	140,728	114,272	**0**	149,711	105,289	149,711	105,289	**0**
	7	180,261	74,74	180,261	74,74	**0,00**	177,205	77,795	177,205	77,795	**0**	175,089	79,911	175,089	79,911	**0**
	Mean (n=3)					**21,04**					**14,577**					**21,73466667**
UCLEVS (LEVs)	1	168,647	86,35	179,998	75,00	**11,35**	177,459	77,541	177,459	77,541	**0**	155,402	99,598	176,691	78,309	**21,289**
	2	152,51	102,49	152,51	102,49	**0,00**	140,728	114,272	140,728	114,272	**0**	149,711	105,289	149,711	105,289	**0**
	3	180,261	74,74	180,261	74,74	**0,00**	177,205	77,795	177,205	77,795	**0**	175,089	79,911	175,089	79,911	**0**
	Mean (n=3)					**3,78**					**0**					**7,096333333**

Figure 3; Supplementary Material 1, Figure S2		Alix					Flotillin-1					THP				
		Alix	inverted	background	inverted	OD	Flottilin-1	inverted	background	inverted	OD	THP	inverted	background	inverted	OD
UC48min	1	111,664	143,336	179,998	75,002	**218,338**	133,636	121,364	178,674	76,326	**45,038**	145,833	109,167	182,069	72,931	**36,236**
	2	89,684	165,316	135,417	119,583	**284,899**	126,715	128,285	161,378	93,622	**34,663**	90,195	164,805	183,594	71,406	**93,399**
	3	120,708	134,292	120,708	134,292	**268,584**	107,303	147,697	159,66	95,34	**52,357**	92,047	162,953	142,405	112,595	**50,358**
	Mean (n=3)					**257,2736667**					**44,01933333**					**59,99766667**
UCLEVS (SEVs)	1	117,133	137,867	179,998	75,002	**62,865**	152,237	102,763	178,674	76,326	**26,437**	136	119	182,069	72,931	**46,069**
	2	135,417	119,583	135,417	119,583	**0**	161,378	93,622	161,378	93,622	**0**	104,63	150,37	183,594	71,406	**78,964**
	7	120,708	134,292	120,708	134,292	**0**	159,66	95,34	159,66	95,34	**0**	98,268	156,732	142,405	112,595	**44,137**
	Mean (n=3)					**20,955**					**8,812333333**					**56,39**
UCLEVS (LEVs)	1	134,655	120,345	179,998	75,002	**45,343**	177,175	77,825	178,674	76,326	**1,499**	138,994	116,006	182,069	72,931	**43,075**
	2	135,417	119,583	135,417	119,583	**0**	161,378	93,622	161,378	93,622	**0**	117,684	137,316	183,594	71,406	**65,91**
	3	120,708	134,292	120,708	134,292	**0**	159,66	95,34	159,66	95,34	**0**	122,14	132,86	142,405	112,595	**20,265**
	Mean (n=3)					**15,11433333**					**0,499666667**					**43,08333333**

Abbreviation: OD – Optical Densitometry value

Supplementary Table S3. Calculated optical densitometry values of Western-blots correspondent to UC48min and UCwash protocols.

Figure 4		CD9					CD81					CD63				
		CD9	inverted	background	inverted	OD	CD81	inverted	background	inverted	OD	CD63	inverted	background	inverted	OD
UC48min	1	112,615	142,39	180,833	74,17	**68,22**	109,668	145,332	177,216	77,784	**67,548**	89,02	165,98	179,536	75,464	90,516
	2	112,795	142,21	185,257	69,74	**72,46**	106,252	148,748	180,068	74,932	**73,816**	181,112	73,888	181,112	73,888	0
	3	106,077	148,92	185,257	69,74	**79,18**	101,457	153,543	178,163	76,837	**76,706**	118,611	136,389	181,112	73,888	62,501
	Mean (n=3)					**73,29**					**72,69**					**51,00567**
UCwash	1	153,233	101,767	180,833	74,17	**27,60**	177,216	77,784	177,216	77,784	**0**	179,536	75,464	179,536	75,464	0
	2	137,172	117,828	185,257	69,74	**48,09**	171,155	83,845	181,146	73,854	**9,991**	181,112	73,888	181,112	73,888	0
	3	155,106	99,894	185,257	69,74	**30,15**	138,301	116,699	178,163	76,837	**39,862**	181,112	73,888	181,112	73,888	0
	Mean (n=3)					**35,27866667**					**16,61766667**					**0**

Figure 4		Alix					Flotillin-1					THP				
		Alix	inverted	background	inverted	OD	Flottilin-1	inverted	background	inverted	OD	THP	inverted	background	inverted	OD
UC48min	1	104,035	150,965	174,536	80,464	70,501	155,498	99,502	178,674	76,326	23,176	90,227	164,773	176,295	78,705	86,068
	2	149,26	105,74	178,818	76,182	29,558	179,49	75,51	179,49	75,51	0	172,038	82,962	177,814	77,186	5,776
	3	102,917	152,083	178,818	76,182	75,901	131,081	123,919	179,49	75,51	48,409	108,565	146,435	177,814	77,186	69,249
	Mean (n=3)					**58,65333333**					**23,86166667**					**53,69767**
UCwash	1	174,536	80,464	174,536	80,464	0	178,674	76,326	178,674	76,326	0	164,475	90,525	176,295	78,705	11,82
	2	178,818	76,182	178,818	76,182	0	179,49	75,51	179,49	75,51	0	177,814	77,186	177,814	77,186	0
	3	153,216	101,784	178,818	76,182	25,602	170,845	84,155	179,49	75,51	8,645	130,517	124,483	177,814	77,186	47,297
	Mean (n=3)					**8,534**					**2,881666667**					**19,70567**

Abbreviation: OD – Optical Densitometry value

Supplementary Table S4. Calculated optical densitometry values of Western-blots correspondent to UC48min and dUC and EXO protocols.

Figure 5		CD9					CD81					CD63				
		CD9	inverted	background	inverted	OD	CD81	inverted	background	inverted	OD	CD63	inverted	background	inverted	OD
UC48min	1	132,087	122,91	181,737	73,26	**49,65**	137,598	117,402	180,845	74,155	**43,247**	134,058	120,942	177,376	77,624	**43,318**
	2	153,056	101,94	181,737	73,26	**28,68**	131,291	123,709	180,845	74,155	**49,554**	108,933	146,067	177,376	77,624	**68,443**
	3	144,908	110,09	175,464	79,54	**30,56**	126,863	128,137	180,845	74,155	**53,982**	165,027	89,973	177,395	77,605	**12,368**
	Mean (n=3)					**36,30**					**48,92766667**					**41,37633333**
dUC	1	159,257	95,74	181,737	73,26	**22,48**	180,845	74,155	180,845	74,155	**0**	177,376	77,624	177,376	77,624	**0**
	2	163,48	91,52	181,737	73,26	**18,26**	178,944	76,056	180,845	74,155	**1,901**	177,376	77,624	177,376	77,624	**0**
	3	175,464	79,54	175,464	79,54	**0,00**	180,845	74,155	180,845	74,155	**0**	177,395	77,605	177,395	77,605	**0**
	Mean (n=3)					**13,58**					**0,633666667**					**0**
EXO	1	181,737	73,26	181,737	73,26	**0,00**	180,845	74,155	180,845	74,155	**0**	177,376	77,624	177,376	77,624	**0**
	2	181,737	73,26	181,737	73,26	**0,00**	180,845	74,155	180,845	74,155	**0**	177,376	77,624	177,376	77,624	**0**
	3	175,464	79,54	175,464	79,54	**0,00**	180,845	74,155	180,845	74,155	**0**	177,395	77,605	177,395	77,605	**0**
	Mean (n=3)					**0,00**					**0**					**0**

Figure 5		Alix					Flotillin-1					THP				
		Alix	inverted	background	inverted	OD	Flottilin-1	inverted	background	inverted	Densitometry	THP	inverted	background	inverted	OD
UC48min	1	127,166	127,834	178,504	76,496	**204,33**	117,66	137,34	175,855	79,145	**58,195**	114,62	140,38	182,008	72,992	**67,388**
	2	133,358	121,642	178,504	76,496	**198,138**	121,952	133,048	175,855	79,145	**53,903**	154,653	100,347	182,008	72,992	**27,355**
	3	157,416	97,584	175,819	79,181	**176,765**	131,063	123,937	176,817	78,183	**45,754**	166,349	88,651	182,442	72,558	**16,093**
	Mean (n=3)					**193,0776667**					**52,61733333**					**36,94533333**
dUC	1	136,433	118,567	178,504	76,496	**42,071**	139,309	115,691	175,855	79,145	**36,546**	182,008	72,992	182,008	72,992	**0**
	2	136,122	118,878	178,504	76,496	**42,382**	152,235	102,765	175,855	79,145	**23,62**	182,008	72,992	182,008	72,992	**0**
	3	175,819	79,181	175,819	79,181	**0**	176,817	78,183	176,817	78,183	**0**	176,948	78,052	182,442	72,558	**5,494**
	Mean (n=3)					**28,151**					**20,05533333**					**1,831333333**
EXO	1	178,504	76,496	178,504	76,496	**0**	175,855	79,145	175,855	79,145	**0**	163,155	91,845	182,008	72,992	**18,853**
	2	142,112	112,888	178,504	76,496	**36,392**	175,855	79,145	175,855	79,145	**0**	105,351	149,649	182,008	72,992	**76,657**
	3	175,819	79,181	175,819	79,181	**0**	176,817	78,183	176,817	78,183	**0**	82,346	172,654	182,442	72,558	**100,096**
	Mean (n=3)					**12,13066667**					**0**					**65,202**

Abbreviation: OD – Optical Densitometry value

Supplementary Table S5. Calculated optical densitometry values of Western-blots correspondent to UC48min and dUC methods.

Figure S3 in Supplementary Material 1		CD9					CD81					CD63					Alix				
		CD9	inverted	background	inverted	OD	CD81	inverted	background	inverted	OD	CD63	inverted	background	inverted	OD	Alix	inverted	background	inverted	Densitometry
UC48min	1	85,675	169,33	236,129	18,87	**150,45**	80,04	174,957	235,112	19,888	**155,069**	71,628	183,372	232,27	22,73	**160,642**	66,966	188,034	236,056	18,944	**206,978**
	2	56,3	198,70	236,129	18,87	**179,83**	64,844	190,156	235,112	19,888	**170,268**	48,952	206,048	232,27	22,73	**183,318**	61,578	193,422	236,056	18,944	**212,366**
	3	146,161	108,84	236,129	18,87	**89,97**	110,069	144,931	235,112	19,888	**125,043**	67,3	187,7	232,27	22,73	**164,97**	52,927	202,073	236,056	18,944	**221,017**
	Mean (n=3)					**140,08**					**150,1266667**					**169,6433333**					**213,4536667**
dUC	1	231,999	23,00	236,129	18,87	**4,13**	235,112	19,888	235,112	19,888	**0**	198,441	56,559	232,27	22,73	**33,829**	217,094	37,906	236,056	18,944	**56,85**
	2	92,817	162,18	236,129	18,87	**143,31**	235,112	19,888	235,112	19,888	**0**	208,904	46,096	232,27	22,73	**23,366**	175,178	79,822	236,056	18,944	**98,766**
	3	236,129	18,87	236,129	18,87	**0,00**	235,112	19,888	235,112	19,888	**0**	98,586	156,414	232,27	22,73	**133,684**	99,395	155,605	236,056	18,944	**136,661**
	Mean (n=3)					**49,15**					**0**					**63,62633333**					**97,42566667**

Figure S3 in Supplementary Material 1		Flotillin-1					THP					Cytochrome C				
		Flottilin-1	inverted	background	inverted	OD	THP	inverted	background	inverted	OD	Cytochron	inverted	background	inverted	OD
UC48min	1	232,253	22,747	232,253	22,747	0	74,574	180,426	211,818	43,182	137,244	244,41	10,59	244,41	10,59	0
	2	168,556	86,444	232,253	22,747	63,697	60,033	194,967	211,818	43,182	151,785	244,41	10,59	244,41	10,59	0
	3	202,513	52,487	232,253	22,747	29,74	58,239	196,761	211,818	43,182	153,579	244,41	10,59	244,41	10,59	0
	Mean (n=3)					**31,14566667**					**147,536**					**0**
dUC	1	232,253	22,747	232,253	22,747	0	207,759	47,241	211,818	43,182	4,059	244,41	10,59	244,41	10,59	0
	2	232,253	22,747	232,253	22,747	0	85,221	169,779	211,818	43,182	126,597	244,41	10,59	244,41	10,59	0
	3	232,253	22,747	232,253	22,747	0	150,096	104,904	211,818	43,182	61,722	244,41	10,59	244,41	10,59	0
	Mean (n=3)					**0**					**64,126**					**0**

Abbreviation: OD – Optical Densitometry value

Supplementary Table S6. Calculated optical densitometry values of Western-blots correspondent to UC48min protocol.

Figure S6 in Supplementary Material 1		CD9					CD81					CD63					Alix				
		CD9	inverted	background	inverted	OD	CD81	inverted	background	inverted	OD	CD63	inverted	background	inverted	OD	Alix	inverted	background	inverted	OD
UC48min	1	97,206	157,79	178,981	76,02	**81,78**	97,64	157,36	156,832	98,168	**59,192**	86,148	168,852	159,423	95,577	**73,275**	114,808	140,192	175,821	79,179	**219,371**
	2	96,212	158,79	178,982	76,02	**82,77**	117,446	137,554	156,832	98,168	**39,386**	141,654	113,346	159,423	95,577	**17,769**	118,221	136,779	175,821	79,179	**215,958**
	3	107,508	147,49	178,983	76,02	**71,48**	124,478	130,522	156,832	98,168	**32,354**	108,416	146,584	159,423	95,577	**51,007**	139,721	115,279	175,821	79,179	**194,458**
	4	144,7	110,30	178,984	76,02	**34,28**	156,832	98,168	156,832	98,168	**0**	159,423	95,577	159,423	95,577	**47,350333**	169,922	85,078	175,821	79,179	**164,257**
	5	112,075	142,93	178,985	76,02	**66,91**	103,549	151,451	156,832	98,168	**53,283**	87,593	167,407	159,423	95,577	**71,83**	111,488	143,512	175,821	79,179	**222,691**
	6	88,331	166,67	178,986	76,01	**90,66**	82,484	172,516	156,832	98,168	**74,348**	72,779	182,221	159,423	95,577	**86,644**	104,409	150,591	175,821	79,179	**71,412**
	7	97,846	157,15	178,987	76,01	**81,14**	87,638	167,362	156,832	98,168	**69,194**	72,63	182,37	159,423	95,577	**86,793**	99,15	155,85	175,821	79,179	**76,671**
	Mean (n=7)					**72,72**					**46,82243**					**62,095476**					**166,4026**

Figure S6 in Supplementary Material 1		Flotillin-1					THP					Lamin A/C					Cytochrome C				
		Flottilin-1	inverted	background	inverted	OD	THP	inverted	background	inverted	OD	Lamin A/C	inverted	background	inverted	OD	Cytochron	inverted	background	inverted	OD
UC48min	1	108,245	146,755	145,006	109,994	**36,761**	113,755	141,245	168,15	86,85	**54,395**	161,585	93,415	161,585	93,415	**0**	169,463	85,537	169,463	85,537	**0**
	2	145,006	109,994	145,006	109,994	**0**	150,264	104,736	168,15	86,85	**17,886**	161,585	93,415	161,585	93,415	**0**	169,463	85,537	169,463	85,537	**0**
	3	145,006	109,994	145,006	109,994	**0**	100,696	154,304	168,15	86,85	**67,454**	161,585	93,415	161,585	93,415	**0**	169,463	85,537	169,463	85,537	**0**
	4	145,006	109,994	145,006	109,994	**0**	168,15	86,85	168,15	86,85	**0**	161,585	93,415	161,585	93,415	**0**	169,463	85,537	169,463	85,537	**0**
	5	139,993	115,007	145,006	109,994	**5,013**	127,731	127,269	168,15	86,85	**40,419**	161,585	93,415	161,585	93,415	**0**	169,463	85,537	169,463	85,537	**0**
	6	102,413	152,587	145,006	109,994	**42,593**	133,712	121,288	168,15	86,85	**34,438**	161,585	93,415	161,585	93,415	**0**	169,463	85,537	169,463	85,537	**0**
	7	122,459	132,541	145,006	109,994	**22,547**	108,262	146,738	168,15	86,85	**59,888**	161,585	93,415	161,585	93,415	**0**	169,463	85,537	169,463	85,537	**0**
	Mean (n=7)					**15,27343**					**39,21143**					**0**					**0**

Abbreviation: OD – Optical Densitometry value
